# Recent Progress in Self‐Supported Metal Oxide Nanoarray Electrodes for Advanced Lithium‐Ion Batteries

**DOI:** 10.1002/advs.201600049

**Published:** 2016-04-15

**Authors:** Feng Zhang, Limin Qi

**Affiliations:** ^1^ Beijing National Laboratory for Molecular Sciences (BNLMS)State Key Laboratory for Structural Chemistry of Unstable and Stable SpeciesCollege of ChemistryPeking UniversityBeijing100871P.R. China

**Keywords:** nanostructure arrays, metal oxides, lithium‐ion batteries, hierarchical structures, 3D porous substrates

## Abstract

The rational design and fabrication of electrode materials with desirable architectures and optimized properties has been demonstrated to be an effective approach towards high‐performance lithium‐ion batteries (LIBs). Although nanostructured metal oxide electrodes with high specific capacity have been regarded as the most promising alternatives for replacing commercial electrodes in LIBs, their further developments are still faced with several challenges such as poor cycling stability and unsatisfying rate performance. As a new class of binder‐free electrodes for LIBs, self‐supported metal oxide nanoarray electrodes have many advantageous features in terms of high specific surface area, fast electron transport, improved charge transfer efficiency, and free space for alleviating volume expansion and preventing severe aggregation, holding great potential to solve the mentioned problems. This review highlights the recent progress in the utilization of self‐supported metal oxide nanoarrays grown on 2D planar and 3D porous substrates, such as 1D and 2D nanostructure arrays, hierarchical nanostructure arrays, and heterostructured nanoarrays, as anodes and cathodes for advanced LIBs. Furthermore, the potential applications of these binder‐free nanoarray electrodes for practical LIBs in full‐cell configuration are outlined. Finally, the future prospects of these self‐supported nanoarray electrodes are discussed.

## 1 Introduction

The energy crisis is currently considered as one of the most important issues in modern society. The ever‐increasing energy demand has been accommodated mainly by various combustion reactions, which continuously stimulates large depletion of the fossil fuels. It has been proposed that the average reserve‐to‐production ratios of world's oil, natural gas, and coal in 2014 are 52.5, 54.1, and 110 years, respectively.^1^ The looming limit to fossil fuels and the implicative concerns including ocean acidification and global warming due to the emissions of greenhouse gases from these combustion sources prompt us to pay attention to the new energy sources. Consequently, renewable energy (water, wind, solar, biological, geothermal energy, etc.) is a potential candidate to replace existing fossil fuels, and extensive effort has been dedicated to developing and building renewable energy technologies including solar power plants, wind farms, and hydroelectric power stations. However, these intermittent facilities are inevitably affected by conditions of topography and climate. Accordingly, the development of sustainable electrochemical energy storage systems featured with consecutive and reliable energy supply is in urgent demand.^2^

**Figure 1:**
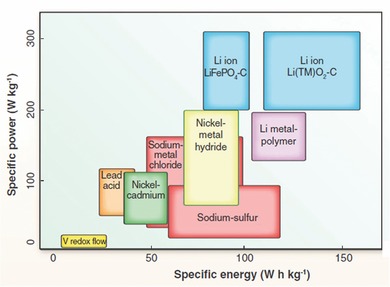
Gravimetric power and energy densities for different commercial rechargeable batteries. Reproduced with permission.^6^ Copyright 2011, American Association for the Advancement of Science.

Rechargeable batteries, as one of the effective electrochemical devices for energy storage, are comprehensively used to power an increasingly diverse range of applications.^3^, ^4^, ^5^ A comparison of different commercial rechargeable batteries in terms of specific energy and power is shown in **Figure** **1**.^6^ Since Sony introduced the first commercial cell in 1991, rechargeable lithium ion batteries (LIBs) have attracted considerable attention because of their appealing advantages such as high energy density, long cycling lifespan, high working voltage, low self‐discharge, and environmental benignity.^7^ Nowadays, their utilization as backup energy supplies has been expanded to a large variety of fields ranging from personal portable electronics (smart phones, laptops, digital cameras, etc.) to electrical vehicles (EVs), hybrid electrical vehicles (HEVs) and smart grids.^8^ In spite of the significant development, LIBs are still fraught with some unsolved problems such as relatively low capacity, poor rate capability, short cyclability, high cost, and potential safe issues. A mobile phone cannot work more than 1–2 days without being recharged; the Tesla Model S offers an industry‐leading driving range over 300 kilometers, which is still inferior to the combustion‐engined motors with a high driving range over 700 kilometers.^9^ The relatively low capacities of LIBs block the way to their long‐range progress. Moreover, the long charging time associated with the poor power density for LIBs cannot satisfy the accelerating pace of society development.^10^ For example, the battery of iPhone 6s (≈1715 mAh in capacity) theoretically takes over 1.5 hours to be fully charged with a standard 1 A USB charger, which is much longer than the ideal charging time (<5 min). Unfortunately, pursuing fast charging rate always brings trade‐offs such as sacrificing the existing capacity or inducing safety hazards.^11^ Hence, advanced LIBs with high rate performance are highly desirable.

Generally, a full LIB consists of four parts: positive and negative electrodes, separator, and electrolyte. Among all the components constituting LIBs, electrode materials are regarded as the key elements dominating the electrochemical performance of LIBs.^12^, ^13^, ^14^ Current commercial LIBs adopt graphite as the anode material and metal oxides or phosphates (such as LiCoO_2_, LiMn_2_O_4_ and LiFePO_4_) as the cathode material. However, the relatively low capacities due to the intercalation mechanism with limited storage of Li^+^ ions usually result in limited energy densities. Particularly, the poor rate capability of these electrode materials makes them work only at a limited range of rates. In other word, once the charging rates are increased to high values, the Li^+^ ions can only stick on the surface rather than insert into the host, leading to considerable capacity decay and potential safety hazards. In this regard, rational design and fabrication of high‐performance nanostructured electrode materials play a significant role in developing next‐generation LIBs with high energy density and power density. As a group of potential alternatives to commercialized electrodes, metal oxides have been comprehensively researched as potential high‐performance electrode materials.^15^, ^16^, ^17^, ^18^ Notably, the fabrication of self‐supported metal oxide nanoarray electrodes without the addition of binders and conductive additives (namely, binder‐free electrodes) provides unique benefits over slurry‐cast electrodes such as enhanced charge transfer efficiency and improved gravimetric capacity. Motivated by their extensive prospects, the design and fabrication of self‐supported metal oxide nanostructure arrays on conductive substrates have aroused extensive interest over last few years.^19^, ^20^, ^21^, ^22^, ^23^ In this regard, various conductive substrates, such as 2D planar substrates and 3D porous substrates, have been employed as the current collectors to construct novel nanoarray electrodes with high performance.

In this review, we summarize the recent progress in the state‐of‐the‐art self‐supported metal oxide nanoarrays with diverse morphologies on 2D/3D conductive substrates as binder‐free electrodes for advanced LIBs, especially with high rate capability. The review is organized as follows: First, the basic fundamentals of metal oxide nanostructures and nanoarrays as LIB electrodes with high capacity and rate capability are outlined and the unique advantages of self‐supported metal oxide nanoarrays are highlighted. Next, the design and fabrication of various metal oxide nanoarrays with different morphologies and architectures, such as 1D nanostructures (e.g., nanowire, nanorod, nanobelt, and nanotube) arrays, 2D nanostructures (e.g., nanosheet, nanoflake, nanoplate, and nanowall) arrays, hierarchical nanostructure arrays, and heterostructured nanoarrays, on 2D/3D conductive substrates are described. Furthermore, some examples of full‐cell LIBs made of self‐supported metal oxide nanoarrays are presented to demonstrate their potential utilizations in practical fields. Finally, the existing challenges and prospects in this research field are discussed.

## 2 Fundamentals of Self‐Supported Metal Oxide Nanoarray Electrodes

### 2.1 Nanostructured Metal Oxides as Electrode Materials for LIBs

The specific capacity of an electrode for LIBs is a key parameter to evaluate the lithium storage performance of the electrode. Practically, gravimetric, volumetric, and areal capacities are used to describe the lithium storage capacity per unit mass, volume, and area, respectively. The theoretical gravimetric capacity is defined as the amount of Li^+^ ions that are able to be stored in per unit mass of the typical material, which can be described by:^24^

(1) $$C_{t}=26800\;\times \frac{n}{M}\;\left(\mathrm{mAh}\;g^{-1}\right)$$

where *C*_t_ in mAh g^–1^ is the theoretical capacity, *n* is the number of electrons in electrochemical reaction, *M* in g mol^–1^ is the molar weight of the specific active material. For the commercialized LiCoO_2_ cathode (the upper voltage of the delithiation of LiCoO_2_ cannot excess 4.2 V vs Li/Li^+^, which means only half of the theoretical capacity of the LiCoO_2_ can be achieved) and graphite anode,^25^ the electrochemical reaction can be denoted as:

(2) $${\mathrm{LiCoO}}_{2}↔\frac{1}{2}{\mathrm{Li}}^{+}+\frac{1}{2}e^{-}{\mathrm{+Li}}_{0.5}{\mathrm{CoO}}_{2}$$

(3) $${\mathrm{Li}}^{+}{\mathrm{+C}}_{6}{\mathrm{+e}}^{-}↔{\mathrm{LiC}}_{6}$$

Then, the theoretical capacities of LiCoO_2_ and graphite are calculated as 137 and 372 mAh g^–1^, respectively. The relatively low capacity undoubtedly handicaps the realization of high energy density. The way of developing high‐performance electrodes with higher capacities is to pack as many Li^+^ ions into the electrodes as possible so that the electrochemical reaction is able to involve a higher stoichiometric ratio of Li^+^ during charging and discharging processes. As a promising alternative to commercial electrodes, metal oxides have long been studied as potential electrode materials for LIBs. In contrast to the intercalation reaction that occurs for LiCoO_2_ and graphite, most of these materials can interact with Li^+^ through conversion/alloying reaction mechanisms. For metal oxides that undergo conversion reactions, such as VO_2_, V_2_O_5_, Fe_2_O_3_, Fe_3_O_4_, Co_3_O_4_, NiO, MnO_2_, and NiCo_2_O_4_, the reaction mechanism can be generalized as follows:

(4) $$M_{x}O_{y}\mathrm{+2}y{\mathrm{Li}}^{+}\mathrm{+2}ye^{-}↔x\mathrm{M+}y{\mathrm{Li}}_{2}O$$

where M represents the transition metal. Besides, some metal oxides, such as SnO_2_, ZnO, and ZnCo_2_O_4_, undergo a further alloying reaction after the initial conversion reaction, as represented below:

(5) $$\mathrm{M+}x{\mathrm{Li}}^{+}+xe^{-}↔{\mathrm{MLi}}_{x}$$

Both equations indicate that metal oxides are able to react with Li^+^ ions with multi‐electron redox reaction. Therefore, high capacity and improved energy density can be predicted when metal oxides act as electrodes in LIBs.

The rate capability related to the characteristic of charging/discharging under high current density is another crucial parameter for evaluating the battery performance. It is undesirable to pursue high capacity at the expense of sacrificing rate capability. To this end, nanostructured metal oxide electrodes have been extensively investigated and utilized in LIBs.^26^, ^27^, ^28^, ^29^ In contrast to the bulk metal oxide electrodes, the nanostructured metal oxide electrodes consisting of structural units in the range of nanometer scale hold numerous virtues including higher specific surface areas, which provide more surfaces accessible for the electrolyte and hence improved electrochemical reaction kinetics, and shortened charge transfer pathways, which make it easier for diffusion of Li^+^ ions and electrons even at large current densities during charge/discharge process. These advantageous features are of great importance for the high rate performance that needs fast charge transfer and enhanced interfacial electrochemical reaction kinetics.

However, the use of metal oxides as LIB electrodes is still baffled by several problems. Firstly, most of the widely studied oxides have the intrinsic nature of high resistance so that they usually demonstrate poor electron conductivity. This phenomenon is unfavorable for improving the rate performance for LIBs because the high resistance of electron transport prevents the combination of electrons and Li^+^ ions, and the electrochemical reaction can not finish thoroughly. Secondly, metal oxides with the conversion/alloying mechanism are inevitable to suffer from large irreversible capacity loss (>30%) during initial cycles, which results from the decomposition of electrolyte and the formation of unstable solid electrolyte intermediate (SEI) layer on the surfaces of metal oxides.^30^ Thirdly, the metal oxides experiencing conversion/alloying reactions often suffer from the severe capacity fading arising from the large volume change during lithiation/delithiation, leading to the poor cycling longevity relating to the pulverization of the electrodes, and even safety problems. Finally, the bulk metal oxides are usually subjected to the poor transport kinetics that seriously slows down the migration of Li^+^ ions. Although metal oxides made into nanoparticles could shorten the diffusion length of Li^+^, the severe aggregation and the increased grain boundaries undoubtedly result in the degenerative cycling and rate performance, and the battery gums up. As a practical solution to these problems, self‐supported metal oxides nanoarrays on conductive substrates represent promising electrodes with high capacity, high rate capability and long cycle life for advanced LIBs.

### 2.2 Self‐supported Metal Oxide Nanoarrays as Binder‐Free Electrodes

**Figure 2:**
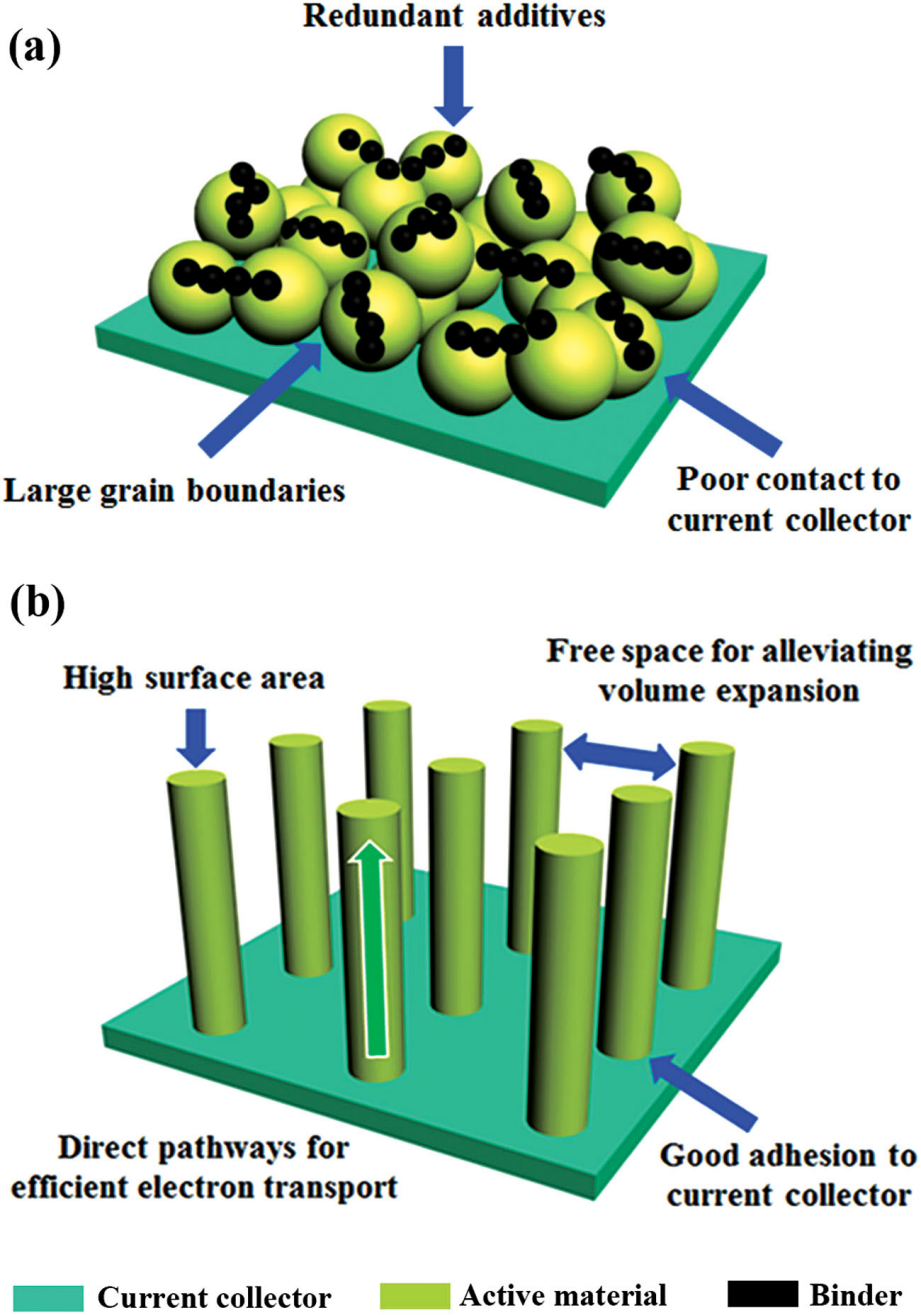
Schematic illustration of internal structure of a) conventional thin film electrode and b) self‐supported nanoarray electrode.

Conventionally, an LIB electrode is made by mixing the powders of electroactive materials with conductive additives and polymer binders to form a slurry, followed by drop‐casting onto a Cu/Al foil and pressing into a thin film.^31^, ^32^ The internal structure of a typical thin film electrode is illustrated in **Figure** **2**a. Such a thin film electrode usually suffers from several drawbacks: Firstly, the large interface electrical resistance resulting from the increased amount of grain boundaries inside the electrode brings about many barriers for electrons and Li^+^ ions to transport. Secondly, the poor contact between active materials and current collector results in large charge transfer resistance that is unfavorable for electron transport, leading to a low efficiency of electron collection. Thirdly, the electrode additives including binders and carbon black have some adverse effects on the specific capacity of the whole electrode. The additives do not interact with Li^+^ ions but their weight ratio in the thin film normally exceeds 20%,^33^ and hence the “dead weight” inside the electrode seriously hinders the improvement of specific capacity. Besides, the insulated binders can increase the internal resistance of electrode and trigger a number of interfaces amid the electron transport path. On the contrary, the growth of the electroactive material directly on a conductive substrate in the form of nanostructure arrays eliminates the need for electrode additives and the additional step of slurry casting during electrode fabrication.^19^, ^20^ This new kind of electrode, namely, self‐supported nanoarray electrode, exhibits many inherent advantages over the conventional slurry‐cast electrode with respect to capacity, rate capability, and cyclability for LIBs (Figure 2b) in addition to the merits of increased gravimetric capacity associated with the binder‐free electrodes:

1. Nanostructured active materials: The time required for the intercalation of lithium ions within an electrode material (*τ 6*) can be expressed as: where *D* represents the diffusion coefficient and *λ* represents the lithium ion diffusion length. Hence, a decrease of the diffusion length for lithium ions by means of particle size minimization is an effective way to improve the rate capability of an electrode material.No matter whether 1D, 2D or hierarchical nanostructures are concerned, the sizes of the individual nanostructures constituting the arrays are within the nanoscale range at least in one dimension. Thus the transport length for Li^+^ ion diffusion inside the electrode is greatly shortened and the mass transfer kinetics is accelerated, thereby significantly improving the high‐rate performance of the electrode. Besides, the nanostructure arrays have large specific surface areas compared with bulk materials. The high contact surface area between electrolyte and active material makes Li^+^ ions more accessible to the active materials and enhances the electrochemical reaction kinetics, thus favoring the improvement of the rate capability.
2. Direct pathways for electrons: The self‐supported metal oxides nanoarrays fabricated directly on 2D/3D conductive substrates hold certain virtues for electron transport. Without the addition of binders, the reduced grain boundaries and decreased interface transfer resistance ensure that the electrons are able to transport along the lattices of electroactive metal oxides, leading to high efficiency of electron transport in the electrode. Moreover, the tight contact between metal oxides and the surface of conductive substrate allows for rapid electron collection and transportation to outer circuit. In this way, even under the circumstances of high discharge/charge current, the battery can work normally and avoid the safety issues arising from inadequate electron transport and large internal resistance of the electrode.
3. Tunable free space inside nanoarrays: Since severe volume expansion of the metal oxides with conversion/alloying mechanism occurs upon lithium insertion, enough free space should be introduced into the interior of the electrode to alleviate this problem. The interspaces between arrayed nanostructures offer sufficient space to relieve the large volume change and severe aggregation of metal oxides, thus avoiding the pulverization of the electrode as well as preventing the nanoarrays from falling off from the substrate. The integrity of the electrode is a prerequisite for achieving stable capacity and enhanced cycling performance. However, excessive free space undoubtedly has adverse effects on advancing tap density as the volumetric capacity will be decreased with increasing free space, and there would be an optimized free space for a specific nanoarray electrode.
4. Controllable morphology of metal oxide nanoarrays: The performance of the nanoarray electrodes is largely dependent on the morphology and structure of the metal oxide nanoarrays. The morphology of metal oxide nanoarrays can be easily modulated by adjusting the synthetic method and growth conditions. In particular, the moderate solution phase approaches have been proved as an effective strategy to regulate the morphology and structural parameters of individual nanostructures as well as their packing density. Moreover, hierarchically structured nanoarrays made of a single metal oxide and heterostructured nanoarrays consisting of composite metal oxides can be fabricated through multi‐step growth/deposition process in a facile way.
5. Tunable structure of conductive substrates: The self‐supported nanoarray electrodes made of metal oxide nanoarrays grown on 2D planar substrates are generally characterized by excellent rate capability and high cyclability, which are potentially applicable for small‐scale energy storage. Nevertheless, the nanoarray electrodes with a planar structure usually suffer from relatively low loading density of the active material and hence limited areal capacity. On the other hand, the 3D porous conductive substrates, such as Ni foam, Cu foam, and graphene foam, represent a novel kind of current collectors to support metal oxide nanoarrays with a high loading density. Moreover, the large amount of micrometer‐ and submicrometer‐sized macropores inside the 3D conductive substrates guarantee fast penetration of electrolyte, thus greatly shortening the activation time of the electrode and the electrochemical reaction time. If the metal oxide nanoarrays grown on the 3D porous substrates are fabricated into hierarchical porous nanostructure arrays, the co‐existing multiple pores (e.g., macropores from the substrates, nanopores from the active materials, and interspaces between arrayed nanostructures) provide a highly open structure for easy diffusion of Li^+^ ions and buffer the large volume change during the charging‐discharging process.

## 3 Self‐Supported Metal Oxide Nanoarrays on 2D Planar Substrates

Initially, the commercial LIBs employ Cu or Al foil as current collector with good conductivity and electrochemical stability in electrodes. Generally, Al foil is used as current collector at cathode and Cu foil is used as current collector at anode considering the alloying effect between metals Li and Al. Thereafter, other 2D planar substrates such as foils of Ti, Ni, Au, stainless steel, and other alloying metals have been developed due to their facile synthesis craft, good conductivity, thermal and electrochemical stability. The choice of the conductive substrates is largely dependent on their stability in the solution systems suitable for the fabrication of specific metal oxide nanoarrays. For example, stainless steel foil with favorable chemical and thermal stability is often utilized under acid/alkali conditions to fabricate nanoarrays through hydrothermal methods. Ti foil has a decent ability to bear acid environment but can be easily corroded in alkali environment. Accordingly, Ti foil is often used as the Ti source under alkali condition for directly constructing Ti‐based metal oxide nanoarrays without additional Ti‐containing reactants. Cu and Ni foils suffer from poor chemical stability in acid environment at high temperatures, but they can be treated as effective substrates in alkali environment. Au foil is the most chemically and thermally stable planar substrate, which is widely utilized under harsh acid/alkali conditions; however, it suffers from high cost. Nowadays, these 2D planar conductive substrates have been frequently employed as current collectors for the controlled growth of various metal oxide nanoarrays, leading to the formation of unique self‐supported nanoarray electrodes with a planar structure for LIBs with improved performance.

### 3.1 1D and 2D Nanoarrays

1D nanostructures, such as nanowires, nanorods and nanotubes, have demonstrated superior structural and physical properties with unique electrical, optical, photovoltaic, and mechanical properties.^34^, ^35^, ^36^ When fabricated on 2D planar conductive substrates in the form of 1D nanostructure arrays, their merits such as shortened diffusion lengths of ions, direct pathways for electron transport, high surface area, sufficient free space between individual nanostructure units, good adhesion between nanoarrays and substrates bring about significant improvement of electrode performance, especially in gravimetric capacity, rate capability, and cyclability.

**Figure 3:**
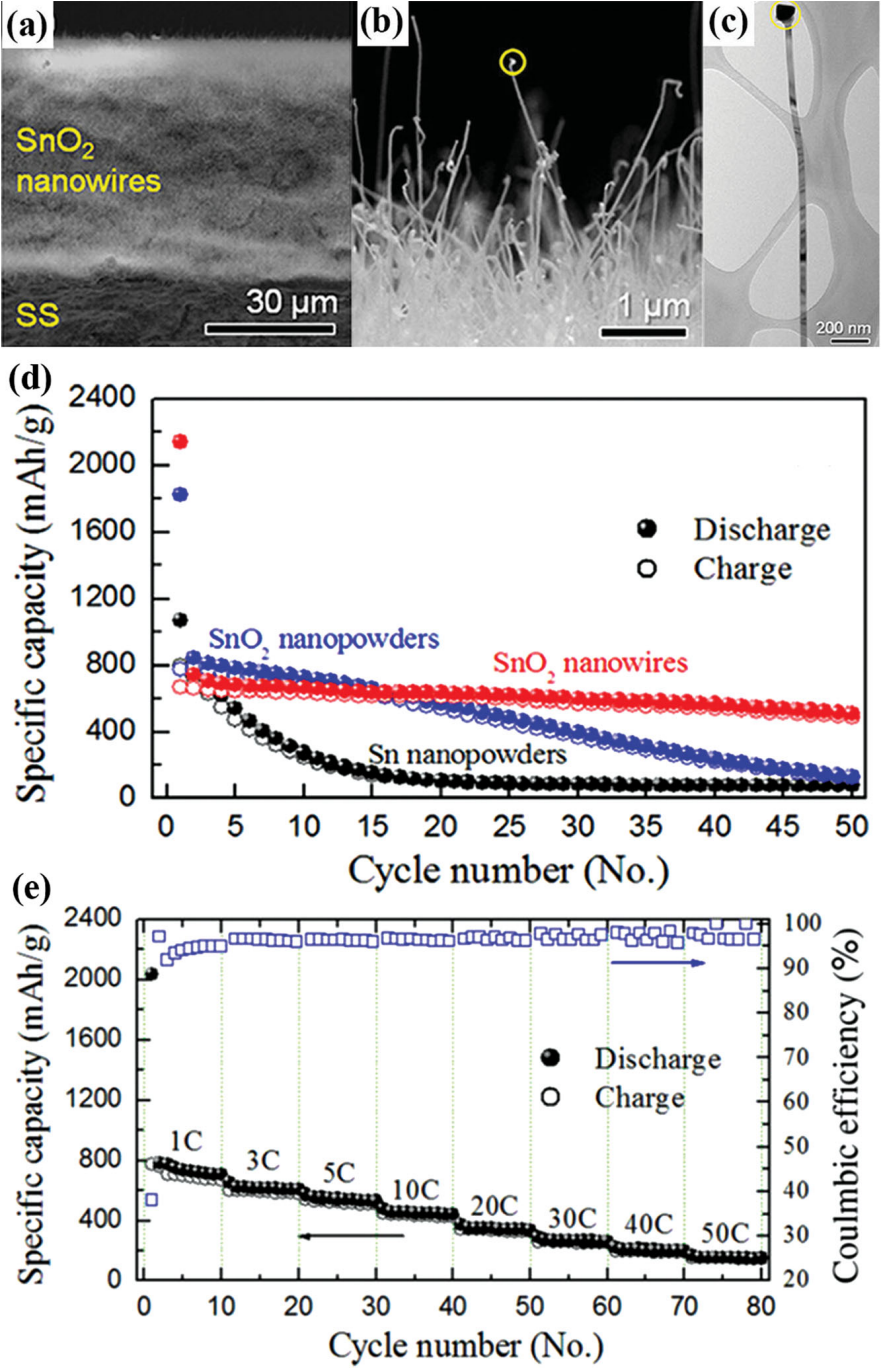
a) Tilted and b) magnified, cross‐sectional SEM images of SnO_2_ nanowires synthesized on stainless steel substrate at 600 ºC. c) TEM image of an individual SnO_2_ nanowire. d) Cycling performance at a current density of 1 C during 50 cycles for three different Sn‐based electrodes. e) Rate performance of SnO_2_ nanowire electrode at different rates and its corresponding Coulombic efficiency. Reproduced with permission.^45^ Copyright 2009, IOP Publishing.

With a theoretical capacity of 790 mAh g^–1^, SnO_2_ has been considered as a potential anode candidate for high capacity LIBs.^37^, ^38^ It may be noted that some works have demonstrated that both the conversion and alloying reactions of SnO_2_ are reversible and so the theoretical capacity for SnO_2_ could achieve 1494 mAh g^–1^.^39^, ^40^, ^41^ Nevertheless, the theoretical capacity of 790 mAh g^–1^ has been adopted for SnO_2_ in most of the previous publications and the reported C‐rates are generally calculated based on this theoretical capacity. For convenience, the value of 790 mAh g^–1^ is considered as the theoretical capacity for SnO_2_ hereafter. Despite a high theoretical capacity, the crucial factors hindering the practical application of SnO_2_ anode include large irreversible capacity loss during the initial cycle, poor ion/electron conductivity, and a large volume expansion up to 300% during lithiation.^42^, ^43^ These problems can be partially solved through the growth of SnO_2_ nanorod arrays directly on conductive substrates. The first two examples of self‐supported SnO_2_ 1D nanostructure arrays used as binder‐free anodes for LIBs were reported in 2009, which were fabricated by hydrothermal growth^44^ and thermal evaporation,^45^ respectively. The SnO_2_ nanorod arrays on Fe‐based alloy substrate were prepared via a facile hydrothermal process.^44^ When tested as an anode for LIBs, the SnO_2_ nanorod arrays exhibited considerably enhanced stability and rate capability in comparison to the disordered nanorods and nanoparticles of SnO_2_. In particular, a capacity of 580 mAh g^–1^, which corresponded to 74.3% of the theoretical capacity of SnO_2_, was retained after 100 cycles at 0.1 C, and a capacity of 350 mAh g^–1^, which was close to the theoretical capacity of graphite, was achieved at high current density of 5 C. Meanwhile, self‐supported SnO_2_ nanowire arrays were fabricated on Au‐catalyzed stainless steel substrate based on vapor–liquid–solid (VLS) mechanism via a thermal evaporation technique.^45^ **Figure** **3**a presents a typical tilted scanning electron microscopy (SEM) image of the as‐prepared SnO_2_ nanowires and Figure 3b shows an enlarged cross‐sectional SEM image revealing the straight and long (over 1 μm at least) morphological characteristic of the SnO_2_ nanowires. The transmission electron microscopy (TEM) image shown in Figure 3c shows the presence of Au catalyst on the tip, which evidenced the VLS growth mechanism for the SnO_2_ nanowires. The collection of specific capacity versus cycle number plots over a voltage window of 0.0–1.2 V at a current density of 1 C during 50 cycles for three different Sn‐based electrodes is shown in Figure 3d. The SnO_2_ nanowires exhibited considerably improved cycling performance with a capacity of 510 mAh g^–1^ at 1 C after 50 cycles, which was superior to those of the SnO_2_ and Sn nanopowder electrodes. It is worth mentioning that the self‐supported SnO_2_ nanowire electrode demonstrated outstanding rate capability with reversible discharge capacities of 600 mAh g^–1^ at 3 C, 530 mAh g^–1^ at 5 C, and 440 mAh g^–1^ at 10 C (Figure 3e). Afterwards, the carbon‐coated SnO_2_ nanorod arrays directly grown on Fe–Co–Ni alloy substrate were produced by a controllable two‐step hydrothermal method for further improvement of the electrochemical performance through carbon coating.^46^ A capacity of around 585 mAh g^–1^ was achieved at 500 mA g^–1^ after 50 cycles, which was superior to the pristine SnO_2_ nanorod arrays with a capacity of only 320 mAh g^–1^ under the same testing condition. Even at a high current density of 3000 mA g^–1^, the capacity of the carbon‐coated SnO_2_ nanorod arrays retained 320 mAh g^–1^.

ZnO, which has a high theoretical capacity of 978 mAh g^–1^, is another metal oxide that has attracted considerable attention as an anode material.^47^ Nevertheless, the poor electron conductive nature and large volume change during the discharge‐charge process result in limited rate performance and cyclability of ZnO‐based electrodes.^48^, ^49^ To give a reasonable solution to these underlying problems, the carbon/ZnO nanorod array electrode was fabricated on Ni foil by a simple carbonization of pre‐adsorbed glucose on ZnO arrays.^50^ The obtained carbon/ZnO nanorod array electrode with a coating layer thickness of 6 nm showed improved cycling performance with a capacity of 330 mAh g^–1^ at 0.25 C after 50 cycles. Also, the remarkable high rate performance of carbon/ZnO nanorod array electrode was demonstrated with a discharge capacity of 360 mAh g^–1^ at 0.75 C after 30 cycles, which was superior to the pristine ZnO array and disordered ZnO nanorods. In addition, a simple chemical bath deposition method was developed for constructing ZnO microrod arrays on copper foil.^51^ When measured as anode in LIBs, the capacity of ZnO microrod arrays decayed only 20% of the initial capacity (over 500 mAh g^–1^) after 100 cycles at 0.5 A g^–1^, and a stable capacity of 220 mAh g^–1^ was obtained at 2 A.

With a relatively high theoretical capacity of 674 mAh g^–1^, CuO has been regarded as another potential anode material for LIBs; unfortunately, the large volume variations (≈174%) during lithiation/delithiation hinder their long‐range utilization.^52^, ^53^, ^54^ Similarly, constructing CuO nanoarrays on conductive substrate provides an effective method to solve this problem. For example, CuO nanoribbon arrays were fabricated on Cu substrate via chemical bath deposition process.^55^ When evaluated as an anode for LIBs, the CuO nanoribbon array electrode showed excellent cyclability with a specific capacity of 608 mAh g^–1^ (89% of the theoretical capacity) retained at the current density of 175 mA g^–1^ after 275 cycles. Furthermore, a capacity of 332 mAh g^–1^ was delivered at 800 mA g^–1^, indicating a good rate capability. As another example, CuO nanorod arrays were fabricated on Cu substrate through in situ conversion of Cu_2_(OH)_3_NO_3_ nanostructures.^56^ The as prepared CuO nanorod arrays were featured with good contact with Cu substrate and sufficient free space between individual CuO nanorods to relax the volume changes. Therefore, the CuO nanorod arrays exhibited good cyclability with a high retained capacity of 650 mAh g^–1^ at 0.5 C and 450 mAh g^–1^ at 2 C after 100 cycles. Additionally, 1D CuO pine‐needle‐like (PNL) arrays were produced directly on Cu substrate via an anodic polarization route.^57^ When used as an anode in LIBs, the CuO PNL arrays demonstrated excellent cyclability with a capacity of 583.1 mAh g^–1^ after 100 cycles at a rate of 2 C and high rate capability with a capacity of 492.2 mAh g^–1^ retained at 20 C.

**Figure 4:**
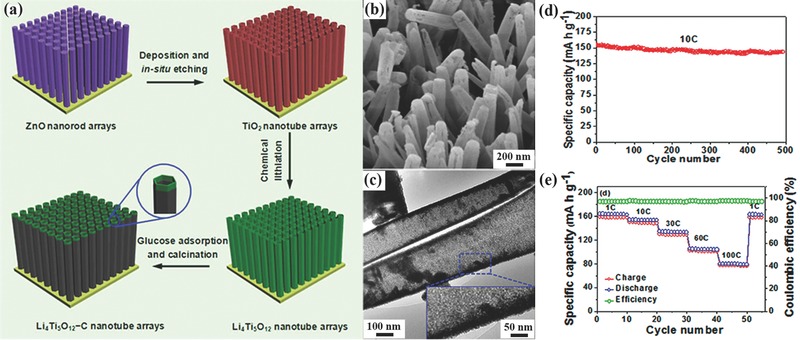
a) Schematic representation of the fabrication of well‐aligned Li_4_Ti_5_O_12_–C nanotube arrays on stainless steel foil from a facile and scalable ZnO template‐based solution method. b) SEM image of Li_4_Ti_5_O_12_–C nanotube arrays. c) TEM image of Li_4_Ti_5_O_12_−C nanotubes exhibiting a wall thickness of approximate 20−30 nm. Inset shows the high‐magnification TEM image of the edge of a single nanotube, clearly displaying the nanoporous wall. d) Cycling performance of Li_4_Ti_5_O_12_–C nanotube arrays at 10 C rate. e) Rate capability of Li_4_Ti_5_O_12_–C nanotube arrays at different rates. Reproduced with permission.^64^ Copyright 2014, American Chemical Society.

As another kind of 1D nanostructures, metal oxide nanotubes can also be fabricated on conductive substrates to prepare self‐supported nanoarrays.^58^, ^59^, ^60^ Compared with 1D nanowire/nanorod arrays, the nanotube arrays not only possess the advantages of shortened ion diffusion length, fast electron transport, and good adhesion between nanoarrays and substrate, but also hold the merits of enhanced electrochemical reaction active sites and specific surface area arising from the unique hollow structure, which is particularly beneficial to the high‐rate performance. The Ti‐based materials have received considerable attention for safe LIBs due to their relatively high operating potential and good thermal stability.^61^ In particular, spinel lithium titanate Li_4_Ti_5_O_12_ (LTO) has been regarded as one of the most promising anode materials for replacing graphite anode.^62^, ^63^ Unlike the metal oxides with the conversion or alloying mechanism for lithium storage, Li_4_Ti_5_O_12_ interacts with Li^+^ ions with an intercalation mechanism, leading to a “zero‐strain” anode material with a theoretical capacity of 175 mAh g^–1^. Although the capacity is lower than that of commercial graphite, the numerous advantages including good compatibility with electrolyte and perfect cycling life due to the zero volume change during lithiation/delithiation are attracting considerable attention. In addition, although the energy density is somewhat sacrificed due to the high potential flat around 1.5 V (vs Li^+^/Li), the SEI layer during the initial intercalation reaction with Li^+^ ions around 0.8 V could be avoided and the safety will be ensured. In this regard, self‐supported Li_4_Ti_5_O_12_‐C nanotube arrays on stainless steel foil were prepared via a facile template‐based solution route.^64^ As shown in **Figure** **4**a, the ZnO nanorod arrays were first constructed on stainless steel foil as template to obtain TiO_2_ nanotube arrays through liquid deposition followed by in situ etching. Then, large‐scale topotactic transformation of the TiO_2_ nanotube arrays to uniform Li_4_Ti_5_O_12_ nanotube arrays was achieved by chemical lithiation with LiOH solution and post annealing at 550 ºC. Finally, the Li_4_Ti_5_O_12_–C nanotube arrays on stainless steel foil were realized by carbonization of glucose adsorbed on the inner and outer surfaces of the Li_4_Ti_5_O_2_ nanotubes. The SEM image shown in Figure 4b reveals densely aligned Li_4_Ti_5_O_12_–C nanotubes with diameters of 200–300 nm. The TEM image shown in Figure 4c suggests that these nanotubes have a uniform carbon shell with a shell thickness of about 20−30 nm, which is composed of dense nanoparticles with sizes of several nanometers. When tested as an anode in LIBs, the Li_4_Ti_5_O_12_–C electrode demonstrated outstanding cyclability and rate capability. A capacity of around 150 mAh g^–1^ was retained at 10 C after 500 cycles (Figure 4d). Remarkably, at high current densities of 30 C, 60 C, and 100 C, reversible and stable capacities could be maintained at 135 mAh g^–1^, 105 mAh g^–1^, and 80 mAh g^–1^, respectively (Figure 4e). The excellent electrochemical performance of the Li_4_Ti_5_O_12_–C nanotube arrays could be attributed to the shortened lithium diffusion distance, high contact surface area, and sufficient conductivity by the introduction of carbon coating layer, and good structure stability of the nanotube arrays.

TiO_2_ is another Ti‐based anode material with intercalation reaction mechanism for advanced LIBs, which has a relatively high theoretical capacity of 335 mAh g^–1^.^65^, ^66^ The fabrication of self‐supported TiO_2_ nanotube arrays directly on metal sheets has been widely investigated.^67^, ^68^ Self‐organized electrochemical anodization is one of the most common methods for constructing TiO_2_ nanotube arrays on metal substrates for the purpose of achieving high areal capacity.^69^, ^70^ For example, a two‐step anodization process followed by annealing was established for fabricating anatase TiO_2_ nanotube arrays on Ti foil.^71^ When used as an anode for LIBs, the TiO_2_ nanotube arrays exhibited a high and stable areal capacity of 0.46 mAh cm^–2^ at a current density of C/10 (1 C = 0.5 mA cm^–2^) after 100 cycles. These TiO_2_ nanotube arrays also showed a remarkable rate capability with an areal capacity of 0.24 mAh cm^–2^ obtained at 5 C.

**Figure 5:**
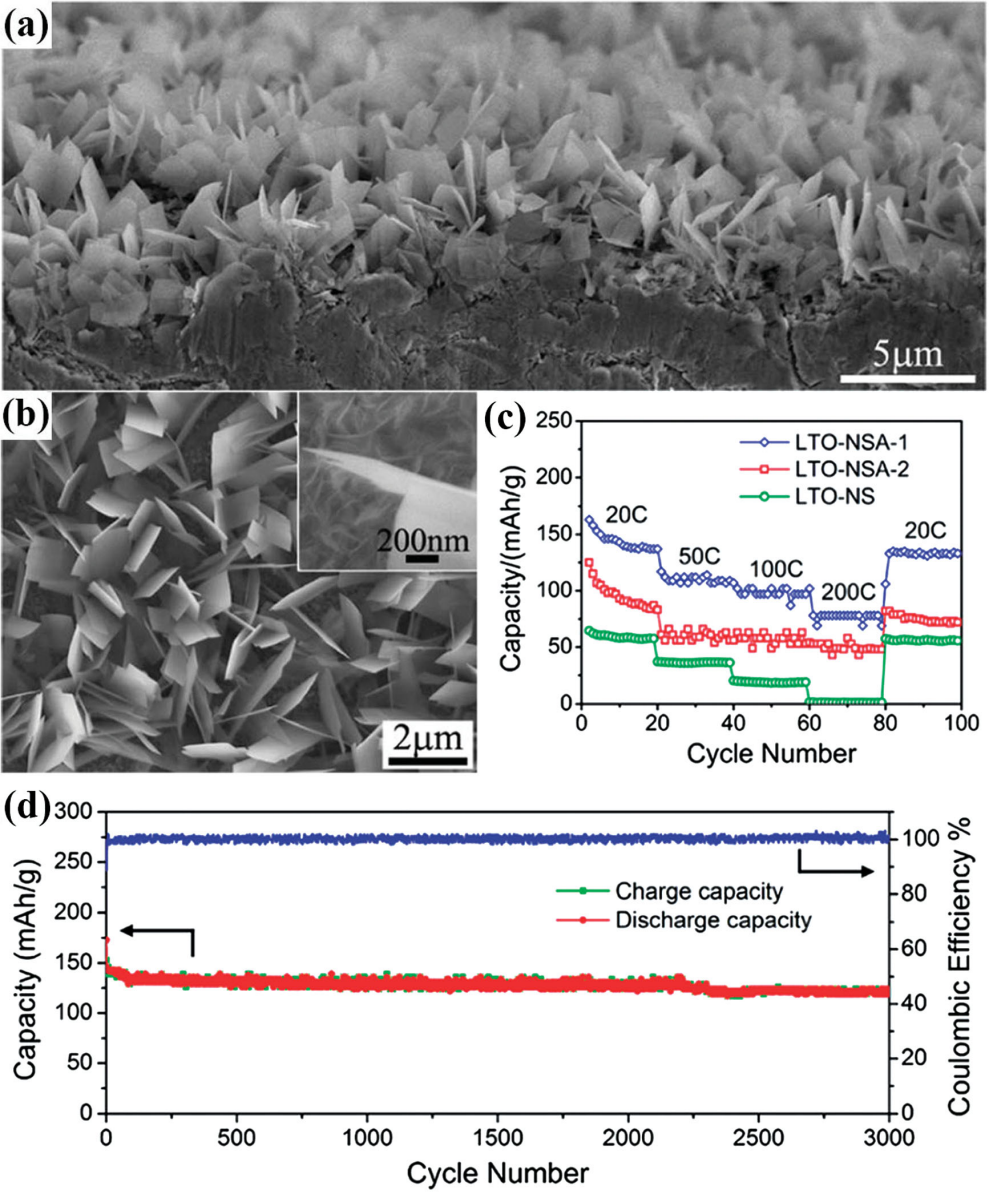
a) Cross‐sectional and b) top‐view SEM images of LTO nanosheet arrays standing on Ti foil. The inset in panel (b) is a high‐magnification image showing the co‐existence of large nanosheets and small nanosheets. c) Rate performance of different LTO nanosheet structures. d) Specific capacity and Coulombic efficiency for 3000 cycles at 50 C for LTO‐NSA‐1. Reproduced with permission.^73^ Copyright 2014, Royal Society of Chemistry.

2D nanostructures such as nanosheets, nanoflakes and nanowalls have aroused considerable attention because they enjoy the mutual merits with 1D nanostructures as electrode materials for LIBs, such as direct pathways for electron transport, shortened pathways for Li^+^ ions diffusion, and high specific surface area offering more Li^+^ transport channels.^72^ Recently, we successfully realized the facile fabrication of well‐aligned Li_4_Ti_5_O_12_ (LTO) nanosheet arrays grown directly on Ti foil through hydrothermal corrosion and growth in LiOH solution followed by topotactic transformation.^73^ The SEM images shown in **Figure** **5**a,b suggest that the vertically aligned rectangular nanosheets are about 1 μm in width and about 2 μm in length. The as‐synthesized LTO nanosheet arrays standing on Ti foil could be directly tested as a binder‐free anode for LIBs. Figure 5c shows the rate performance of different LTO nanosheet structures including self‐supported arrays of LTO nanosheets ≈14 nm in thickness (LTO‐NSA‐1), self‐supported arrays of LTO nanosheets ≈80 nm in thickness (LTO‐NSA‐2), and randomly dispersed LTO nanosheets (LTO‐NS), which suggests that the self‐supported nanosheets arrays are superior to the randomly dispersed nanosheets. It is noteworthy that the LTO‐NSA‐1 anode exhibited an excellent rate capability with a reversible capacity of 163 mAh g^–1^ at 20 C and a capacity of 78 mAh g^–1^ retained even at a very high rate of 200 C (≈18 s to full charge). Significantly, the LTO‐NSA‐1 anode cycled at a rate of 50 C reached a stable capacity of 134 mAh g^–1^ within 100 cycles, and retained a capacity of 124 mAh g^–1^ after 3000 cycles with a capacity loss of only 7.5% (Figure 5d). The ultralong cyclability and outstanding rate capability of the Li_4_Ti_5_O_12_ nanosheet arrays could be attributed to the good contact with current collector and the structural stability that prevented collapse and aggregation of nanosheets.^74^ Furthermore, the large exposed surface area and the thin thickness of the Li_4_Ti_5_O_12_ nanosheets ensured the better contact for electrolyte and improved ion diffusion kinetics. Interestingly, a flexible LIB, which could be fully recharged within 30 s and was able to light an LED, was assembled by using the LTO nanosheet arrays standing on Ti foil as the bendable anode.

NiO is another representative anode material for LIBs with a high theoretical capacity of 718 mAh g^–1^. The vertically aligned NiO nanowall electrode on Ni foil was created by a simple plasma assisted oxidation method.^75^ The nanoarray electrode consisting of individual NiO nanowalls with a thin thickness less than 40 nm showed good cyclability. A stable capacity of 638 mAh g^–1^ at 1.25 C after 85 cycles was delivered. Only 3% capacity loss between 2 and 85 cycles was discovered, indicating the unique NiO nanowall morphology with the small wall thickness brought in lots of merits for facilitating electron/ion transport and good adhesion between active materials and current collector. Even at high rate of 1.87 C, a capacity of 490 mAh g^–1^ was achieved. Later on, single‐crystalline NiO nanoflake arrays grown directly on copper substrates were fabricated by a modified hydrothermal synthesis and post‐annealing.^76^ The capacity of the nanoflake array anode was retained at 720 mAh g^–1^ over 20 cycles at a current density of 100 mA g^–1^. Even at a high rate of 1 A g^–1^ (1.5 C), it was capable of retaining a capacity of 500 mAh g^–1^ over 40 cycles.

SnO_2_ nanosheets represent a promising candidate for anode materials in LIBs.^77^ Recently, Lou and co‐workers developed a facile template‐free solution method combined with a post annealing treatment to grow interconnected SnO_2_ nanosheets on various conductive substrates.^78^ Moreover, a second layer of SnO_2_ nanosheets were grown on top of the first layer by simply repeating the procedure to achieve a higher loading mass of the active material. When tested as binder‐free electrodes for LIBs, the electrodes delivered reasonably high specific capacity, good cycling stability and good rate capability. Although the single‐layer and double‐layer SnO_2_ nanosheet samples demonstrated a similar gravimetric capacity, the double‐layer sample exhibited a reversible areal capacity of 260 μAh cm^–2^ after 30 cycles at 200 mA g^–1^, which was much higher than that for the single‐layer sample (111 μAh cm^–2^). The double‐layer SnO_2_ nanosheet arrays showed a good rate capability with a discharge capacity of 400 mAh g^–1^ at the current density of 1500 mA g^–1^.

While extensive research on the fabrication of self‐supported metal oxide nanoarrays on 2D conductive substrates as binder‐free anodes has been carried out, there are only few reports on the fabrication of self‐supported metal oxide nanoarrays on 2D substrates as cathodes for LIBs. This may be partially ascribed to the limited kinds of metal oxides suitable for LIB cathodes and the difficulties in fabricating the nanostructure arrays of complex metal oxides containing multiple metals directly on conductive substrates. Vanadium oxides with a wide range of oxidation states of vanadium from +2 as in VO to +5 as in V_2_O_5_ have been regarded as an attractive material for energy applications.^79^ As a potential candidate as cathode for LIBs, V_2_O_5_ with a high theoretical capacity of 294 mAh g^–1^ based on the intercalation mechanism of two Li^+^ ions has drawn considerable attention; however, the poor conductivity for both of ion and electron, unsatisfying structural stability, and sluggish electrochemical kinetics considerably hinder their future development.^80^ As an example of nanoarray cathodes for LIBs, V_2_O_5_ nanoarrays on Ti substrate with different structural units were fabricated through calcination of the vanadium hydrate precursors.^81^ For the V_2_O_5_ nanobelt arrays, a stable and high capacity of 255 mAh g^–1^ was delivered after 50 cycles at 50 mA g^–1^ with only a slight decay of 10%. Spinel LiMn_2_O_4_ is a commercial cathode material for LIBs with a theoretical capacity of 148 mA g^–1^. Recently, unique LiMn_2_O_4_ nanorod arrays on Pt foil were prepared by lithiation of α‐MnO_2_ nanotube arrays in molten salts followed by 800 ºC annealing in air.^82^ The as prepared LiMn_2_O_4_ nanorod arrays showed outstanding cycling performance with a reversible capacity of 113 mAh g^–1^ after 200 cycles at 1 C, corresponding to an average capacity loss of about 0.06% per cycle. Furthermore, a high discharge capacity of 106 mAh g^–1^ that retained 81% of full capacity was achieved at 10 C, demonstrating good high‐rate performance of the LiMn_2_O_4_ nanorod arrays.

### 3.2 Hierarchical Nanoarrays

Hierarchical structures exhibiting structural features at different size scales are generally characterized by ordered assemblies of building blocks or subunits. Hierarchical structures comprising nanoscale building blocks show promising prospects for LIB electrode materials because small particle size, high specific surface area, and other structural features including accessible porous systems and large void spaces can be readily introduced to a hierarchical structure through self‐assembly of nanoscale subunits.^83^ Herein, hierarchical nanoarrays refer to aligned arrays of hierarchical 1D and 2D nanostructures (e.g., nanorods, nanobelts, nanotubes, nanosheets, nanoplates, and nanowalls) consisting of nanoscale building blocks. For these hierarchical porous nanostructure arrays, in addition to the merits associated with small particle size and large surface area, the free spaces and pores between subunits are able to offer accessible surfaces for electrolytes and alleviate the volume expansion during lithium insertion.

**Figure 6:**
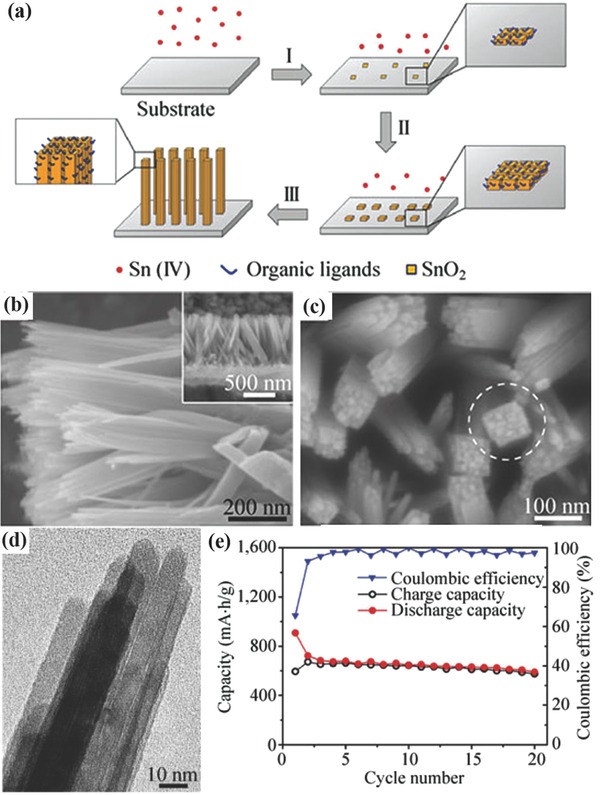
a) Schematic illustration of a tentative mechanism for the growth of mesocrystalline SnO_2_ nanorod arrays on a substrate. b) Cross‐sectional and c) top‐view SEM images of mesocrystalline SnO_2_ nanorod arrays. Inset in panel (b) shows a low‐magnification image. d) TEM image of the end of a single mesocrystalline SnO_2_ nanorod. e) Charge/discharge capacity and Coulombic efficiency of mesocrystalline SnO_2_ nanorod arrays cycled at 10 C. Reproduced with permission.^86^ Copyright 2013, Springer.

Mesocrystals, which are crystallographically oriented nanoparticle superstructures with high crystallinity and high porosity, have been demonstrated to be promising electrode materials for LIBs.^84^, ^85^ We established a general method for facile kinetics‐controlled growth of aligned arrays of mesocrystalline SnO_2_ nanorods on arbitrary substrates by adjusting supersaturation in a unique ternary solvent system.^86^ The possible formation mechanism of the mesocrystalline SnO_2_ nanorod arrays is outlined in **Figure** **6**a. In the early stage, the nucleation of SnO_2_ only occurred on the substrate by carefully controlling the supersaturation (stage I). Meanwhile, the organic ligands were adsorbed on the surface of the initial SnO_2_ nuclei and then wrapped in nucleating crystals, forming initial mesocrystalline nuclei. As the hydrolysis processes progressed, the initial SnO_2_ nuclei grew in height and width, evolving into well‐defined, tetragonal SnO_2_ nanorods consisting of a bundle of primary nanorods (Stage II). Then, the nanorod subunits in the mesocrystalline nuclei grew independently along the specific direction and finally formed mesocrystalline nanorod arrays with organic ligands filled in the interspaces to prevent the fusion of subunits (Stage III). The obtained SnO_2_ nanorod arrays had a length of ≈750 nm and average diameter of ≈80 nm, and the square nanorods were actually made of bundles of primary nanorods (Figure 6b,c). This TEM image shown in Figure 6d indicates the presence of parallel primary nanorods ≈10 nm in width with some interspaces between them. Owing to the unique combination of the mesocrystalline structure and the 1D nanoarray structure, the obtained mesocrystalline SnO_2_ nanorod arrays grown on Ti substrate exhibited a superior rate performance when used as an anode material for LIBs. At a current rate as high as 10 C (i.e., 7820 mA g^–1^), the mesocrystalline nanoarray electrode exhibited an excellent reversible discharge capacity of 720 mAh g^–1^ and retained 590 mAh g^–1^ after 20 cycles (Figure 6e).

Co_3_O_4_ is a typical transition metal oxide with a high theoretical capacity of 890 mAh g^–1^ as an LIB anode material and its practical applications are also hindered by the large volume change during the lithiation/delithiation process and the capacity loss during the first cycle.^87^ A mild chemical bath method was developed for the growth of large‐area, self‐supported Co_3_O_4_ nanowire arrays on Ti foil.^88^ The pore produced by the topotactic transformation from Co(OH)_2_ to Co_3_O_4_ during the growth process resulted in unique hierarchical porous nanowires. The self‐supported Co_3_O_4_ nanowire arrays delivered a capacity of 700 mAh g^–1^ after 20 cycles at 1 C (1 C = 111 mA g^–1^, which was defined as one Li^+^ per formula in an hour), which was superior to the non‐self‐supported Co_3_O_4_ nanowires (350 mAh g^–1^) and the commercial Co_3_O_4_ powders (80 mAh g^–1^). Besides, the self‐supported Co_3_O_4_ nanowire arrays exhibited excellent high‐rate performance with specific capacities of 450 mAh g^–1^ and 240 mAh g^–1^ retained at 20 C and 50 C, respectively. Afterwards, mesoporous Co_3_O_4_ nanobelt arrays on Ti foil were fabricated by a facile hydrothermal method with subsequent transformation process.^89^ The as‐prepared Co_3_O_4_ nanobelt arrays showed a capacity of 770 mAh g^–1^ at 1.5 C (1 C = 111 mA g^–1^) after 25 cycles, and a capacity of 330 mAh g^–1^ at 30 C after 30 cycles, indicating good cycling performance and excellent rate capability. Later on, Co_3_O_4_ porous rhombus‐shaped nanorod arrays^90^ and hierarchical nanopeapod arrays^91^ were successfully fabricated and they also exhibited superior lithium storage performance.

Another representative Co‐based oxide is CoO, which has a theoretical capacity of 716 mAh g^–1^ as an LIB anode material.^92^, ^93^ Porous CoO nanowire arrays were prepared on Ti substrate via a hydrothermal process with subsequent pyrolysis.^94^ The CoO nanowires consisted of large quantities of pores resulting from the release of gases during the pyrolysis of precursor process. When tested as an anode in LIBs, the CoO porous nanowire arrays delivered a relatively high capacity of 670 mAh g^–1^ at 1 C after 20 cycles. Reversible capacities of 464 mAh g^–1^, 312 mAh g^–1^, and 150 mAh g^–1^ were retained at current densities of 2 C, 4 C, and 6 C, respectively. Recently, CoO nanowire clusters (NWCs) on copper current collector were fabricated and evaluated as the anode of binder‐free LIBs.^95^ The produced CoO nanowires were composed of nanoparticles with a diameter of 10 nm, indicating a hierarchical structure. When applied as a binder‐free anode in LIBs, the CoO NWCs showed high capacity of 1248.8 mAh g^–1^ at the current density of 1 C after 50 cycles, while the conventional electrode prepared by non‐self‐supported CoO NWCs showed a sharply capacity decay with a capacity of 440 mAh g^–1^ after 50 cycles at 1 C. Furthermore, the rate performance of self‐supported CoO NWCs was also satisfying with a capacity of 1330.5 mAh g^–1^ maintained at 5 C. The observation of a specific capacity larger than the theoretical capacity could be attributed to two aspects: First, the pseudo‐capacitive effect and the double‐layer effect played a vital role for the additional capacity. Second, the nanosized Li_2_O produced during the charging/discharging process could act as an oxidizer for the conversion of Co^2+^ to Co^3+^, thus storing more Li^+^ ions in the electrode. In addition, a SiO_2_ coating shell was introduced to the CoO NWCs to produce CoO@SiO_2_ NWCs with improved cycling performance. As expected, the inner CoO nanowires could only expand into voids between nanoparticles under the SiO_2_ shell protection, and a longer cycle life was successfully achieved with a capacity over 1200 mAh g^–1^ retained after 200 cycles at 1 C.

α‐Fe_2_O_3_ is a promising candidate for high‐performance LIB anodes because of its high theoretical capacity (≈1000 mAh g^–1^), non‐toxicity, high abundance, high corrosion resistance, and low processing cost.^96^ However, α‐Fe_2_O_3_ anodes usually suffer from poor cyclability caused by the drastic volume expansion (>100%) during lithiation and the performance degradation at high current densities associated with the low conductivity. Self‐supported α‐Fe_2_O_3_ nanoarrays have received considerable attention due to their unique properties and potential applications in energy conversion and storage. In particular, porous α‐Fe_2_O_3_ nanorod arrays were fabricated on Ti foil via a facile hydrothermal method followed by annealing.^97^ The obtained α‐Fe_2_O_3_ nanorods were composed of numerous pores due to the dehydration and the lattice contraction during the thermal decomposition of FeOOH. When investigated as an anode in LIBs, the porous α‐Fe_2_O_3_ nanorod arrays exhibited good cyclability with a high capacity of 562 mAh g^–1^ retained after 50 cycles at 134.2 mA g^–1^. A reversible capacity of 459 mAh g^–1^ was achieved at 2 C. Recently, we reported the fabrication of self‐supported hierarchical α‐Fe_2_O_3_ nanorod arrays consisting of mesocrystalline nanorod bundles with tunable interstices by hydrothermal growth coupled with chemical etching.^98^ The hydrothermal growth of mesocrystalline α‐Fe_2_O_3_ nanorods directly on Ti foil was achieved in acetic acid solutions, which avoided the step of transformation from FeOOH, and the chemical etching was implemented to realize the structural optimization of the α‐Fe_2_O_3_ nanorod arrays. The etched α‐Fe_2_O_3_ nanorods with optimized interspaces demonstrated remarkable electrochemical properties when evaluated as anode in LIBs. A high capacity of 950 mAh g^–1^ was achieved after 100 cycles at 1 C while the nanorod arrays without etching showed a rapid capacity loss with a capacity of 210 mAh g^–1^ retained after 40 cycles at 1 C. Meanwhile, the etched α‐Fe_2_O_3_ nanorod arrays showed excellent rate capability with a capacity of 970 mAh g^–1^ retained at high current density of 5 C after 130 cycles. Even at higher rate of 30 C, an acceptable capacity of 350 mAh g^–1^, which was close to that for the commercial graphite anode, was maintained.

**Figure 7:**
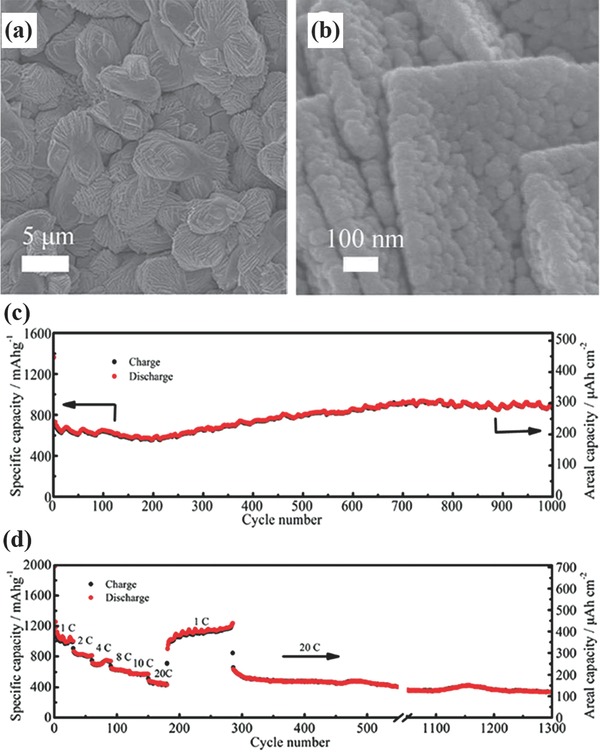
a,b) SEM images of 3D hierarchical porous α‐Fe_2_O_3_ nanosheets. c) Cycling performance at 2 C and d) rate capability of binder‐free 3D hierarchical porous α‐Fe_2_O_3_ nanosheet electrode. Reproduced with permission.^100^

While α‐Fe_2_O_3_ nanoflakes were prepared on Cu foil by using a thermal treatment method,^99^ hierarchical porous α‐Fe_2_O_3_ nanosheets on Cu foil were recently prepared by a hydrothermal growth followed by precursor conversion.^100^] **Figure** **7**a shows a representative SEM image of the α‐Fe_2_O_3_ nanosheet arrays obtained through conversion from the precursor FeOOH nanosheet arrays. The enlarged SEM image shown in Figure 7b suggests that each nanosheet with a porous structure comprised of numerous interconnected nanoparticles, indicating a unique hierarchical porous structure. When tested as a binder‐free anode in LIBs, the porous α‐Fe_2_O_3_ nanosheet array electrode exhibited outstanding cycling performance with a capacity of 850 mAh g^–1^ (301.5 μA h cm^–2^) at 2 C after 1000 cycles (Figure 7c). The long cycle life could be attributed to the more exposed active sites and a stronger adhesion to the substrate, which ensured the integrity of the whole electrode without serious damage. Figure 7d displays the rate capability of the hierarchical porous α‐Fe_2_O_3_ nanosheet array electrode, which shows that a discharge capacity of 433.2 mAh g^–1^ (152 μA h cm^–2^) was delivered at a high current rate of 20 C. The outstanding rate capability could be related to the formation of a conductive network made of metallic Fe arising from the irreversible reactions during cycling to enable good electron transfer. Similarly, porous α‐Fe_2_O_3_ nanosheets grown on Ti foil were produced via solvothermal synthesis followed by post‐annealing.^101^ The as‐prepared hierarchical α‐Fe_2_O_3_ nanosheets consisted of large amounts of interconnected nanoparticles and exhibited highly porous texture with plenty of nanopores throughout the nanosheets. When investigated as an anode in LIBs, the α‐Fe_2_O_3_ nanosheet arrays showed good cycling performance with a capacity of 908 mAh g^–1^ at 100 mA g^–1^ after 60 cycles, and a good rata capability with a capacity of 573 mAh g^–1^ retained at 2000 mA g^–1^. In addition, mesoporous CuO nanosheet cluster arrays (MNCAs) were fabricated on Cu foil through an ammonia vapor‐phase corrosion method.^102^ The obtained mesoporous MNCAs had a high surface area and exhibited enhanced electrochemical performance. A high specific capacity of 639.8 mAh g^–1^ was delivered after 100 cycles at 1 C rate and a reversible capacity of 548.8 mAh g^–1^ was maintained at a high rate of 10 C.

Spinel ternary transition metal oxides have attracted considerable interest owing to the complementarity and synergy of the two transition metal oxides during charge and discharge processes. The ternary metal oxides usually exhibit better electrical conductivity and higher electrochemical activity compared with mono‐metal oxdies. Moreover, the abundant redox reactions of the ternary metal oxides during Li^+^ ions insertion/extraction give rise to high specific capacity (≈1000 mAh g^–1^) for an anode material. However, the huge volume expansion during lithiation often leads to poor cycling performance. Engineering spinel ternary metal oxides into hierarchical nanoarrays is an effective way to obtain high‐performance anodes for LIBs. For example, hierarchical NiCo_2_O_4_ nanorod arrays on Cu foil were realized through a template‐free hydrothermal deposition and a subsequent annealing process.^103^ When tested as an anode in LIBs, a capacity of 830 mAh g^–1^ was achieved at 0.5 C (1 C = 860 mA g^–1^) after 30 cycles, which is surperor to that of Co_3_O_4_ nanorod arrays with a capacity 727 mAh g^–1^ under the same testing condition. The rate capability of hierarchical NiCo_2_O_4_ nanorod arrays was also impressive with a capacity of 127 mAh g^–1^ retained at 110 C. Besides, hierarchical Co_x_Mn_3–x_O_4_ arrays were grown on conductive stainless steel by a facile solvothermal route and a subsequent annealing treatment.^104^ The morphology of the final products could be adjusted from hierarchical CoMn_2_O_4_ nanowires to MnCo_2_O_4_ nanosheets by simply controlling the volume ratio of water and ethanol. The CoMn_2_O_4_ nanowire arrays exhibited good cycling performance with a capacity of 450 mAh g^–1^ at 800 mA g^–1^ after 30 cycles, and good rate capability with a capacity of 215 mAh g^–1^ retained at 10 C (1 C = 700 mA g^–1^). The MnCo_2_O_4_ nanosheet arrays demonstrated a good capacity of 460 mAh g^–1^ at 800 mA g^–1^ after 30 cycles, and exhibited good rate capability with 270 mAh g^–1^ retained at 10 C (1 C = 700 mA g^–1^). In addition, hierarchical mesoporous Cu_x_Co_3–x_O_4_ nanosheet arrays were directly fabricated on Ti foil by a facile hydrothermal method and a subsequent annealing treatment.^105^ The product showed a high capacity of 1107 mAh g^–1^ at 400 mA g^1^ after 50 cycles, and a high rate capability with 335 mAh g^–1^ retained at 50 C (1 C = 1100 mA g^–1^).

There are only few reports on the fabrication of hierarchical metal oxide nanostructure arrays as binder‐free cathodes for LIBs. LiCoO_2_ is a commercial cathode material for LIBs that has been extensively investigated.^106^, ^107^ Recently, chain‐like high‐temperature LiCoO_2_ (HT‐LiCoO_2_) nanowire arrays on various substrates were successfully fabricated through an in situ lithiation followed by calcination.^108^ First, Co_3_O_4_ nanowire arrays were grown on the substrate by a common hydrothermal and post‐calcination reaction. Then, the lithiation of the Co_3_O_4_ nanowire arrays was performed in LiOH solution by another hydrothermal reaction to obtain low‐temperature LiCoO_2_ (LT‐LiCoO_2_) nanowire arrays with a quasi‐spinel structure. After a further calcination, the HT‐LiCoO_2_ nanowire arrays with a stable spinel structure were obtained. The resultant chain‐like HT‐LiCoO_2_ nanowire arrays with a highly porous structure showed superior cycling and rate performance compared to the LT‐LiCoO_2_ nanowire arrays. There was only 10% decay of capacity after 50 cycles at 0.1 C (1 C = 148 mA g^–1^) for the HT‐LiCoO_2_ nanowire arrays, while 32% capacity decay was observed for the LT‐LiCoO_2_ nanowire arrays under the same conditions. Moreover, the HT‐LiCoO_2_ nanowire arrays delivered a capacity of ≈103 mAh g^–1^ at 10 C, indicating a good rate capability. The enhanced electrochemical performance for the HT‐LiCoO_2_ nanowire arrays could be attributed to the 1D nanowire structure facilitating electron transport and the small LiCoO_2_ grains shortening the diffusion length of Li^+^ ions.

### 3.3 Heterostructured Nanoarrays

Although the nanoarrays composed of a single metal oxide have been demonstrated to be a promising candidate for LIB electrodes with improved cyclability and rate capability, these single metal oxide nanoarrays usually suffer from some problems related to the inherent characteristics of the specific metal oxide. For example, as a typical metal oxide with an intercalation mechanism for lithium storage, Li_4_Ti_5_O_12_ has negligible volume expansion during lithiation and hence exhibits excellent cycling performance, but it has a low theoretical capacity (175 mAh g^–1^), which still impedes its wide utilization in practical life. On the other hand, a lot of metal oxides with the conversion or alloying mechanism for lithium storage usually exhibit high theoretical capacities but they usually suffer from large volume expansion upon lithiation, which leads to poor cyclability. Besides, the low loading mass of metal oxide nanoarrays on the 2D planar substrate always brings about a relatively low specific areal capacity as well as a low energy density. Hence, the strategy of utilization and combination of different metal oxides into an integrated electrode with complementary and/or synergistic effects has received considerable attention for offering a good solution to the problems associated with the single phase metal oxide.^109^, ^110^, ^111^ Particularly, heterostructured nanoarrays composed of composite metal oxides have shown great potential in generating high‐performance nanoarray electrodes for LIBs.

**Figure 8:**
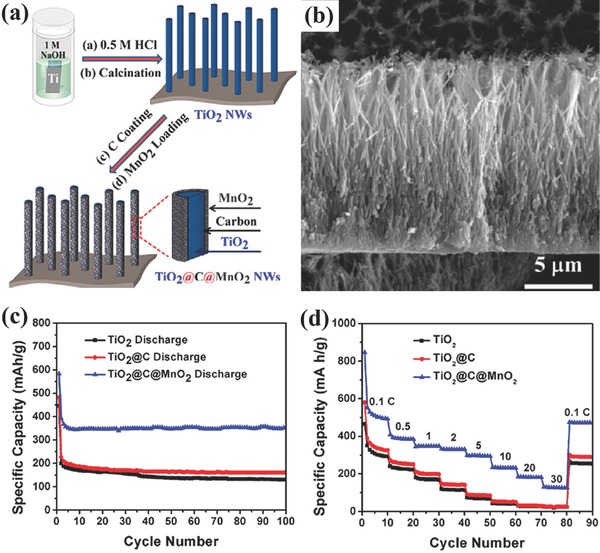
a) Schematic illustration for the fabrication of TiO_2_−C/MnO_2_ core−double‐shell nanowire arrays on Ti foil. b) Cross‐sectional SEM images of TiO_2_−C/MnO_2_ nanowires. c) Cycling performance of three different nanowire electrodes beyond 100 cycles at a rate of 1 C. d) Rate performance of three different nanowire electrodes at various current densities from 0.1 to 30 C. Reproduced with permission.^117^ Copyright 2013, American Chemical Society.

TiO_2_ nanoarrays have been frequently utilized as an effective scaffold with high Li‐insertion potential and negligible volume expansion during lithiation to load other metal oxides for compensating their low gravimetric capacities (the theoretical capacity calculated by one Li^+^ per TiO_2_ is 335 mAh g^–1^) and low areal capacities.^112^, ^113^, ^114^, ^115^, ^116^ For example, unique TiO_2_‐C/MnO_2_ core‐double‐shell nanowire arrays on Ti foil were fabricated as a heterostructured nanoarray anode for LIBs, and the fabrication process is shown in **Figure** **8**a.^117^ TiO_2_ nanowires on Ti foil were first prepared by alkali hydrothermal reaction and ion exchange with HCl, followed by calcination. Then, the carbon coating on TiO_2_ nanowires was accomplished via a glucose‐assisted hydrothermal treatment and subsequent heat treatment. Finally, MnO_2_ nanoparticles were decorated onto the TiO_2_‐C nanowire arrays through immersion into KMnO_4_ aqueous solution at room temperature. Cross‐sectional SEM image of the TiO_2_‐C/MnO_2_ nanowire arrays with a mean length of 12 μm is shown in Figure 8b. The combination of the excellent cycle stability of TiO_2_, high capacity of MnO_2_ (a high theoretical capacity of 1230 mAh g^–1^) and high electronic conductivity of graphitic carbon interlayer resulted in considerably improved cycling and rate performance. The TiO_2_‐C/MnO_2_ nanowire arrays delivered a discharge capacity of 352 mAh g^–1^ after 100 cycles at 0.1 C with a decay of only 10% of the initial discharge capacity, indicating superior cyclability compared with TiO_2_ (130 mAh g^–1^) and TiO_2_‐C (162 mAh g^–1^) nanowire arrays (Figure 8c). The TiO_2_‐C/MnO_2_ nanowire arrays also exhibited improved rate capability with a capacity of 130 mAh g^–1^ at a high rate of 30 C, as shown in Figure 8d. This enhanced electrochemical performance could be ascribed to the unique 1D core−double shell structure with fast Li^+^ ion transportation throughout the electrode, high electronic conductivity and mechanical protection of the carbon layer, and high capacity of the MnO_2_ active material.

On the other hand, the introduction of anatase TiO_2_ to spinel Li_4_Ti_5_O_12_ is also an effective way to improve the low specific capacity of Li_4_Ti_5_O_12_.^118^ For example, dual phase Li_4_Ti_5_O_12_–TiO_2_ nanowire arrays on Ti foil were fabricated as an integrated electrode through a LiOH lithiation of H_2_Ti_2_O_5_·H_2_O nanowire arrays.^119^ The dual phase Li_4_Ti_5_O_12_–TiO_2_ nanowire arrays with a Li_4_Ti_5_O_12_ to TiO_2_ ratio of 2:1 showed improved rate capability over single phase TiO_2_ and Li_4_Ti_5_O_12_ nanowire arrays. Although the specific capacity of single phase TiO_2_ nanoarrays is higher than Li_4_Ti_5_O_12_–TiO_2_ nanoarrays due to the higher theoretical capacity of anatase TiO_2_ compared with Li_4_Ti_5_O_12_ at a low rate of 0.1 C (1 C = 175 mAh g^–1^), the single phase TiO_2_ nanoarrays delivered a capacity of 46.8 mAh g^–1^ at a high rate of 30 C, which was much lower than that of the Li_4_Ti_5_O_12_‐TiO_2_ nanoarrays (115.5 mAh g^–1^). The enhanced rate capability could be attributed to the increased grain boundary densities embedded in the composite, which enhanced the structural stability and lithium storage kinetics by reducing the diffusion time of both electrons and Li^+^ ions. Besides, the zero volume change during lithiation/delithiation of both Li_4_Ti_5_O_12_ and TiO_2_ ensured the structural integration, thus largely improving the cycling performance. As a result, a stable capacity 129.3 mAh g^–1^ at 10 C after 100 cycles was achieved with only 2.5% loss of initial capacity.

Furthermore, the combination of two high capacity metal oxides also showed great potential in achieving high gravimetric or areal capacity.^120^, ^121^, ^122^ For instance, heterostructured SnO_2_/α‐Fe_2_O_3_ nanotube arrays on stainless steel substrate was prepared by using ZnO nanowire arrays as an in situ sacrificial template.^123^ The final composite nanotube arrays exhibited a high areal capacity of 1.289 mAh cm^–2^ at a current rate of 0.1 mA cm^–2^, which were much larger than those of many previous thin‐film/3D microbattery electrodes. The composite nanotube arrays also showed improved cyclability with areal capacities of 0.727 mAh cm^–2^ and 0.344 mAh cm^–2^ retained at 0.1 mA cm^–2^ and 0.3 mA cm^–2^ after 50 cycles, respectively, which were better than that for either single SnO_2_ nanotube arrays or α‐Fe_2_O_3_ nanotube arrays. Moreover, a high discharge capacity of 0.507 mAh cm^–2^ at a high rate of 0.6 mA cm^–2^ was observed, indicating superior rate capability compared with other SnO_2_ nanotube arrays. The outstanding electrochemical performance of the SnO_2_/α‐Fe_2_O_3_ nanotube arrays may be attributed to the the existence of an elegant synergistic effect. Particularly, alloying and dealloying is the dominant process contributing to lithium storage and delivery for SnO_2_ after the first cycle, which occurs at a lower potential as compared to the electrochemical reaction of α‐Fe_2_O_3_ with Li. When SnO_2_ nanoparticles are electrochemically engaged at low potential, α‐Fe_2_O_3_ nanoparticles are almost inactive and can function as a buffering matrix to alleviate the huge strain stress, as well as a block to prevent against generated Sn aggregating. At high potential, SnO_2_ can also help to buffer the volume change of α‐Fe_2_O_3_. In addition, the transition metal Fe produced during the lithiation reaction of α‐Fe_2_O_3_ had a catalytic function to promote the first backward reaction of SnO_2_.^124^, ^125^

## 4 Self‐supported Metal Oxide Nanoarrays on 3D Porous Substrates

**Figure 9:**
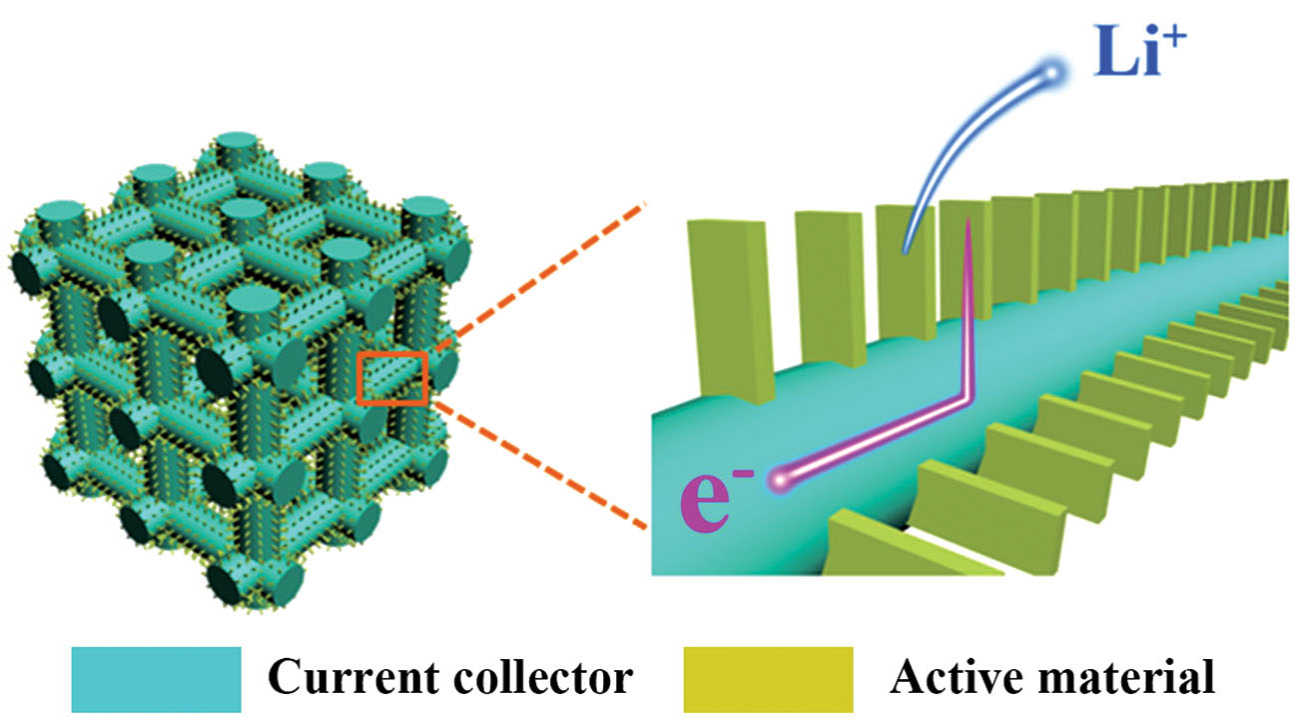
Schematic illustration of self‐supported nanoarrays on a 3D porous substrate.

Self‐supported metal oxide nanoarrays grown on 3D porous conductive substrates as integrated electrodes for LIBs are attracting increasing interest because of their remarkable electrochemical performance. In comparison to the 2D planar substrates, the 3D porous substrates have the following advantages (**Figure** **9**): First, the unique 3D structure provides more growth sites, thus a higher loading mass of active materials can be realized, leading to a higher specific areal capacity and a higher gravimetric capacity with respect to the whole electrode.^126^, ^127^ Second, the unique porous structure of 3D conductive substrates offers accessible pathways for the penetration of electrolyte, accelerating the Li^+^ ion diffusion and interface reaction kinetics.^128^ Finally, the fast electron transport along the 3D conductive substrate is beneficial for improving the high‐rate performance. Therefore, the self‐supported metal oxide nanoarrays on 3D conductive substrates, such as Ni foam, Cu foam, graphene foam, and carbon cloth have been regarded as a promising candidate for advanced electrodes in next‐generation LIBs. Ni and Cu foams have similar virtues such as high conductivity, large porosity, and high mechanical strength. However, they can not endure acid environment at high temperatures. Besides, their overall mass is often much higher than that of active materials, thus impairing the gravimetric capacity in terms of the whole electrode. Graphene foam obtained by replicating Ni foam template inherits the favorable characteristics of Ni foam such as high conductivity and large porosity. It is noteworthy that the lightweight feature owing to the relatively low density of carbon can bring about improved gravimetric capacity of the whole electrode. However, the poor mechanical strength of graphene foam would lead to the collapse of the skeleton structure, resulting in relatively poor cycling performance. Carbon cloth is another representative 3D porous substrate with high conductivity. Moreover, the pore size of carbon cloth is smaller than Cu, Ni, and graphene foams, which gives rise to higher tap density and volumetric capacity of the electrode. Furthermore, the high mechanical strength resulting from the tight twine between interconnected carbon fibers allows for applications in flexible and stretchable devices. However, it has a larger weight compared with graphene foam, which would restrict the gravimetric capacity of the whole electrode.

### 4.1 1D and 2D Nanoarrays

Self‐supported arrays of SnO_2_ nanorods (25 nm in diameter) grown on Ni foam were prepared via a simple hydrothermal method.^129^ A capacity of 607 mAh g^–1^ at 0.2 C (1 C = 782 mA g^–1^) up to 50 cycles was delivered, which was superior to those with diameters of 60 nm and 100 nm. However, the drastic decay of capacity due to the huge volume change still limits their down‐to‐earth applications. As a possible solution to this problem, heterogeneous branched core‐shell SnO_2_–polyaniline (PANI) nanorod arrays were fabricated on Ni foam through hydrothermal growth followed by electrodeposition techniques to realize improved cycling and rate performance.^130^ A discharge capacity of 506 mAh g^–1^ at 200 mA g^–1^ was achieved after 100 cycles while a discharge capacity of 450 mAh g^–1^ at a higher rate of 600 mA g^–1^ was delivered after 50 cycles. The heterogeneous branched core‐shell SnO_2_–PANI nanorod arrays also exhibited outstanding rate capability with a discharge capacity of 312 mAh g^–1^ retained at 3000 mA g^–1^. The introduction of the highly conductive PANI coating shell not only offered strong adhesion with SnO_2_ nanorods for the structure stability and mechanical integrity, but also provided fast electron transport, which contributed to the significant improvement of the electrochemical performance in terms of cyclability and rate capability. As a further effort, an integrated SnO_2_–PPy electrode comprising SnO_2_ nanorod arrays with entire polypyrrole (PPy) coverage on Ni foam were successfully prepared.^131^ The resultant SnO_2_–PPy nanofilm showed much enhanced cyclability with a stable capacity of 701 mAh g^–1^ at 200 mA g^–1^ after 300 cycles and a capacity of 500 mAh g^–1^ at 600 mA g^–1^ after 300 cycles. Even at high rate of 3000 mA g^–1^, a retained capacity of 512 mAh g^–1^ was obtained, indicating the excellent rate capability of the SnO_2_–PPy nanofilm. The outstanding cycling stability and high rate capability could be attributed to the flexible confinement of PPy nanofilm as “buffer agent” for the SnO_2_ nanorod arrays, which maintained the entire structure integrity. Furthermore, the synergetic effect of conducting polymer and Ni foam facilitated 3D electron transport, thus enhancing the rate capability.

In addition to 1D metal oxide nanoarrays, a variety of 2D metal oxide nanoarrays grown on 3D conductive porous substrates have been fabricated as high‐performance anodes for LIBs. For example, Li_4_Ti_5_O_12_ nanosheet arrays on 3D graphene foam (LTO/GF) were fabricated via a simple hydrothermal process.^132^ When investigated as a binder‐free anode in LIBs, the LTO/GF electrode showed not only excellent cycling stability with a specific capacity around 150 mAh g^–1^ at 30 C (1 C = 175 mAh g^–1^) and 130 mAh g^–1^ at 100 C after 500 cycles, but also an outstanding rate performance with a specific capacity of 86 mAh g^–1^ at 200 C (corresponding to an 18 s full discharge). Moreover, 2D nanoarrays of a variety of metals oxides with high theoretical capacities, such as α‐Fe_2_O_3_,^133^ MnO_2_,^134^, ^135^ SnO_2_,^136^ NiO,^137^ have been grown on 3D porous substrates as binder‐free anodes for LIBs. Specifically, interconnected α‐Fe_2_O_3_ nanowall arrays (NWAs) on Ni foam were produced by α‐FeOOH transformation.^133^ When tested as a binder‐free anode in LIBs, the α‐Fe_2_O_3_ NWAs electrode delivered a capacity of 518 mAh g^–1^ at 0.1 C (1 C = 1007 mA g^–1^) after 50 cycles and exhibited enhanced rate capability with reversible capacities of 563 mAh g^–1^ and 440 mAh g^–1^ at 2 C and 5 C, respectively. As another example, interconnected MnO_2_ nanoflake arrays assembled on 3D graphene foam (MnO_2_ NFs@GF) were produced through a facile hydrothermal method.^135^ The obtained MnO_2_ NFs@GF showed an increased cycling performance with a capacity of nearly 1200 mAh g^–1^ after 300 cycles (the current density was 0.2 A g^–1^ in the initial 3 cycles and 0.5 A g^–1^ in the following cycles) and improved rate capability with a capacity over 500 mAh g^–1^ at 5 A g^–1^. In addition, 2D SnO_2_ nanosheet arrays on Ni foam were prepared by a hydrothermal route.^136^ These SnO_2_ nanosheets were interconnected and formed into a highly open and porous structure, which is desirable for the penetration of electrolyte and Li^+^ ions. The SnO_2_ nanosheet arrays showed an improved cycling performance with a discharge capacity of 679.4 mAh g^–1^ at 0.5 C (1 C = 782 mA g^–1^) after 50 cycles, and the rate capability was excellent with a capacity of over 400 mAh g^–1^ at 5 C.

**Figure 10:**
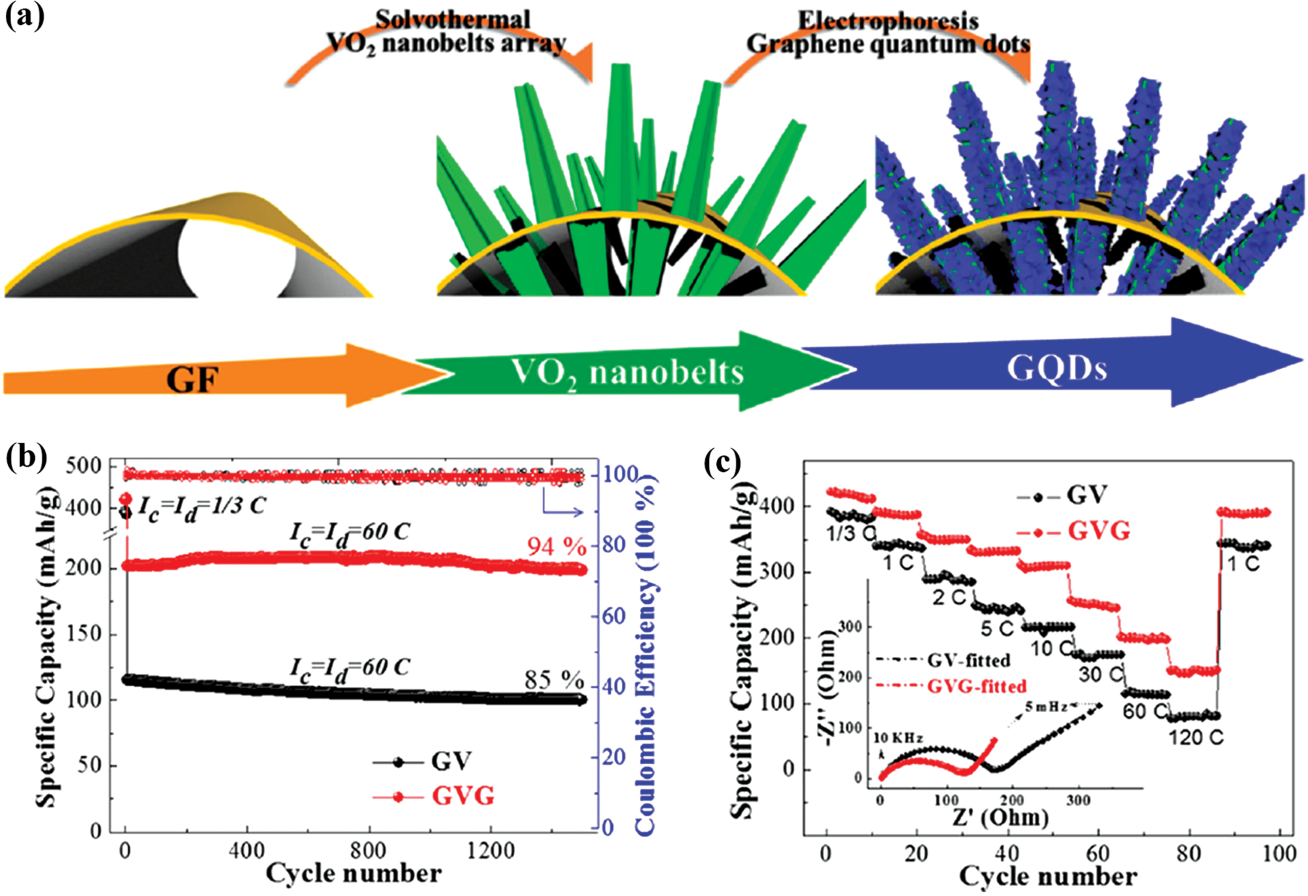
a) Schematic illustration of the fabrication process of GF supported GQDs‐coated VO_2_ nanobelts array. b) Cycling performance of GV and GVG at 60 C for 1500 cycles (1/3 C at first five cycles for activation). c) Rate performance of GV and GVG electrodes. Inset shows AC impedance plots at the full‐charged state after the first cycle. Reproduced with permission.^139^ Copyright 2015, American Chemical Society.

Vanadium oxides have been regarded as promising cathode materials for LIBs owing to their high capacity, low cost, and abundant sources. In particular, VO_2_ has attracted considerable attention because of its unique VO_2_ (B) bilayers, rapid lithium ion diffusion rate and higher capacity than other types of vanadium oxides.^138^ However, the VO_2_ electrode materials usually suffer from fast capacity fading and poor high‐rate performance, probably due to self‐aggregation, dissolution, and the fast increased charge transfer resistance during cycles. Recently, Fan's group reported the fabrication of graphene quantum dots‐anchored VO_2_ nanobelt arrays on 3D graphene foam (GVG) via solvothermal and electrophoresis processes.^139^ The synthesis procedure of the GVG electrode is illustrated in **Figure** **10**a. A CVD‐grown graphene foam (GF) was employed as the substrate to grow VO_2_ nanobelt arrays (denoted as GV) via a solvothermal process. Then homogeneous graphene quantum dots (GQDs) were coated on the VO_2_ nanoarrays by an electrophoresis method. The resultant GVG electrode demonstrated excellent cycling performance with a capacity retention of 94% of the original capacity after 1500 cycles at 60 C (1 C = 300 mA g^–1^) with 1/3 C at first cycles for activation, which was superior to the GV electrode with the capacity retention of 85%, as shown in Figure 10b. Moreover, the rate capability of the GVG electrode was impressive with a capacity of 151 mAh g^–1^ at a high rate of 120 C (Figure 10c). The enhanced electrochemical performance of GVG could be rationalized by considering that the homogeneous GQDs coating can effectively separate the VO_2_ nanobelts from each other and thus avoid agglomeration as well as minimize the dissolution of active materials. Meanwhile, the coating layer of GQDs made the VO_2_ surface more lipophilic, thus enhancing the electrochemical reaction kinetics. As another example of VO_2_ nanoarrays on GF substrate, Fan's group reported the fabrication of VO_2_ nanoflake arrays grown on graphene foam with a shell of hydrogen molybdenum bronze (GF + VO_2_/HMB).^140^ The HMB with both high electrical conductivity and ionic conductivity brought about improved cycling and rate performance. The high capacities of 305 mAh g^–1^ and 209 mAh g^–1^ were achieved after 500 cycles at 5 C and 30 C, respectively. Impressively, the GF + VO_2_/HMB electrode exhibited excellent high‐rate capability with a specific capacity of 280 mAh g^–1^ at 10 C and 219 mAh g^–1^ at 30 C, respectively, which were much higher than those for many commercial cathode materials.

Fan's group also reported the preparation of a V_2_O_5_/conductive polymer (PEDOT) core‐shell hierarchical nanobelt arrays on ultrafine graphene foam (UGF‐V_2_O_5_/PEDOT).^141^ The “arrow‐tail”‐like V_2_O_5_ nanobelts offered desirable merits especially in shorter Li^+^ ion diffusion lengths and direct electron transport. When a mesoporous PEDOT coating layer, the electron transfer was facilitated and the integration of the electrode was preserved. When investigated as a cathode in LIBs, the UGF‐V_2_O_5_/PEDOT electrode showed excellent cyclability and rate capability with a stable specific capacity of 265 mAh g^–1^ at 5 C (1 C = 300 mA g^–1^) after 500 cycles and 163 mAh g^–1^ at 60 C after 1000 cycles. It is worth noting that V_2_O_5_ nanoflake arrays were also fabricated on carbon cloth by a simple solvothermal method.^142^ The electrode demonstrated good cycling performance with a capacity of 275 mAh g^–1^ at 0.5 C (1 C = 300 mA g^–1^) after 100 cycles. The rate capability is characterized by a capacity of 181 mAh g^–1^ retained at 10 C.

### 4.2 Hierarchical Nanoarrays

**Figure 11:**
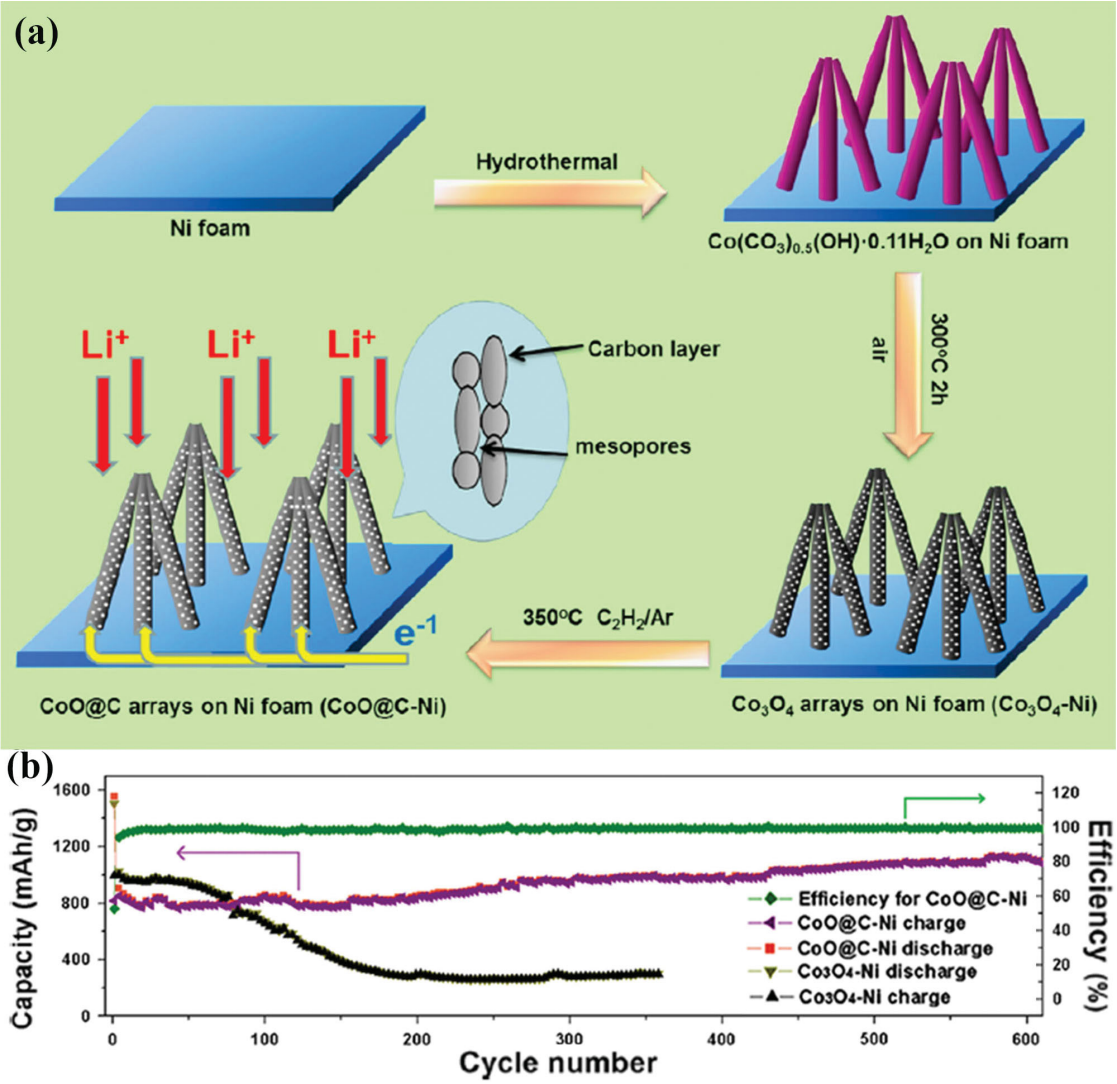
a) Schematic illustration of the synthesis of CoO@carbon metasequoia‐like nanowire arrays on nickel foam (CoO@C–Ni) composites. b) Cycling performance of CoO@C–Ni and Co_3_O_4_–Ni at a rate of 1 A g^–1^. Reproduced with permission.^144^ Copyright 2014, Elsevier.

Self‐supported hierarchical Co_3_O_4_ 1D nanoarrays with a lemongrass‐like morphology were fabricated on Ni foam substrate by a simple hydrothermal synthesis and subsequent calcination.^143^ The nanoarrays were composed of porous 1D nanoblades consisting of small nanoparticles. When investigated as an anode in LIBs, the Co_3_O_4_ nanoarray electrode retained a high reversible capacity of up to 981 mAh g^–1^ after 100 cycles at a rate of 0.5 C (1 C = 890 mA g^–1^) and a capacity higher than 381 mAh g^–1^ even at a rate as high as 10 C. The superior rate capability and reversibility capacity could be ascribed to the following reasons: (1) The lemongrass‐like morphology of Co_3_O_4_ nanoparticles limited the mobility and agglomeration of the particles during cycling, thus enduring the volume changes during lithiation/delithiation; (2) the small nanoparticles of nanoblades shortened the diffusion length of lithium ions, hence benefiting the structural stability and rate capability.Furthermore, hierarchical metasequoia‐like CoO@C nanowire arrays grown on Ni foam (CoO@C‐Ni) was prepared by hydrothermal growth followed by low‐temperature chemical vapor deposition (CVD).^144^ The synthesis process of the hierarchical 1D nanoarrays is schematically illustrated in **Figure** **11**a. The starting precursor Co(CO_3_)_0.5_(OH)·0.11H_2_O metasequoia‐like nanowire arrays were first prepared via traditional hydrothermal treatment of Ni foam. Subsequently, the Co_3_O_4_ nanowire arrays were fabricated on Ni foam by a two‐step annealing process. Then, a unique low‐temperature (300–350 ºC) CVD technique was applied for achieving CoO nanowire arrays and simultaneous introduction of carbon layer. The CoO@C nanowires were composed of interconnected nanoparticles (5–20 nm), resulting in a hierarchical porous structure. When tested as an anode in LIBs, the CoO@C–Ni electrode exhibited excellent cycling stability with a high capacity of 1120 mAh g^–1^ retained after 600 cycles at the current density of 0.1 A g^–1^, which was much better than that of Co_3_O_4_–Ni nanowire arrays with a severe decrease of capacity from 1020 to 280 mAh g^–1^ only after 250 cycles (Figure 11b). Even at a higher rate of 4 A g^–1^, the CoO@C–Ni electrode maintained a capacity of 536 mAh g^–1^, indicating an improved rate capability. The much enhanced electrochemical performance of the CoO@C–Ni hetero‐composites could be attributed to two reasons: First, the introduction of outer thin carbon layer offered improved electrical conductivity and prevented adjacent active materials from contacting each other as a buffer. Second, the 3D oriented‐orderly porous structure was beneficial for the Li^+^ ion diffusion to active sites with less resistance and could remit the volume variations during lithium insertion/extraction.

Hierarchical 1D nanoarrays of various metal oxides other than cobalt oxides on 3D porous substrates have also been fabricated as binder‐free anodes for LIBs. For example, CuO porous nanowire arrays were assembled directly on Cu foam by a simple anodic electrodeposition method.^145^ The CuO nanowires consisting of small nanoparticles showed a highly porous character with numerous diffusion channels accessible to the electrolytes. When tested as anode in LIBs, a high capacity of 687.4 mAh g^–1^ was delivered at 0.15 mA cm^–2^ after 120 cycles. Moreover, CuO/C core/shell nanowire arrays consisting of porous CuO nanowires were fabricated on Ni foam.^146^ Capacities of 610 mAh g^–1^ and 360 mAh g^–1^ were achieved at 0.5 C (1 C = 674 mA g^–1^) and 3 C after 290 cycles, respectively, indicating improved electrochemical performance. Furthermore, hierarchical NiO nanorod arrays anchored on Ni foam were prepared via anodization and subsequent thermal annealing.^147^ The hierarchical NiO nanoarrays composed of many nanocrystals delivered capacities of 705.5 mAh g^–1^ and 548.1 mAh g^–1^ at 1 A g^–1^ and 2 A g^–1^ after 70 cycles, respectively. Even at a high current density of 5 A g^–1^, a relatively high capacity of 575 mAh g^–1^ was obtained.

In addition to the hierarchical 1D nanoarrays, hierarchical 2D nanoarrays on 3D porous substrates have been reported and applied as advanced anodes for LIBs. In particular, a variety of porous cobalt oxide nanosheet arrays on 3D porous substrates have been reported.^148^, ^149^, ^150^ For example, ultrathin and highly ordered 2D CoO nanosheet arrays (NSAs) on Ni foam were prepared by a facile galvanostatic electrodeposition technique.^150^ The CoO NSAs exhibited a good cycling stability with a reversible capacity of nearly 1000 mAh g^–1^ retained after 100 cycles at 1 A g^–1^. They also showed a remarkable rate capability with a high capacity of 560 mAh g^–1^ under at high current density of 10 A g^–1^. The excellent electrochemical performance should be associated with the designed morphology of the CoO NSAs, which had a good solid contact with highly conducting 3D Ni foam that made the electrons rapidly conduct back and forth from the CoO nanoparticles to the current collector. Meanwhile, the formation of the uniform nanopores in the hierarchical CoO nanosheets could provide a highly electrochemically active surface area that facilitated electrolyte diffuse into the inner of the CoO nanosheets, and lots of elastic buffer space to accommodate the volume changes during the lithium insertion/extraction.

Moreover, mesoporous MnO_2_ nanosheet arrays grown on Ni foam were produced via electrodeposition with a subsequent annealing process.^151^ The MnO_2_ nanosheets were interconnected and developed into highly open and porous structure. Meanwhile, the MnO_2_ nanosheets consisted of small pores with the diameter of 8 nm. When tested as an anode in LIBs, the MnO_2_ nanosheet arrays demonstrated excellent cycling performance with a high and stable capacity of ≈900 mAh g^–1^ at 1000 mA g^–1^ after 200 cycles. Additionally, interconnected mesoporous NiO nanosheet arrays were fabricated on Ni foam via a facile solvothermal growth and subsequent calcination.^152^ The nanosheet arrays were featured with interconnected character, and each NiO nanosheet comprised many pores with a diameter of 5 nm. When investigated as an anode in LIBs, a high discharge capacity of 1043 mAh g^–1^ was achieved at 0.2 C (1 C = 718 mA g^–1^) after 80 cycles. Under a high current density of 10 C, a capacity of 305 mAh g^–1^ was retained, indicating good high‐rate performance. As another example, hierarchical mesoporous NiO nanosheet arrays were directly grown on carbon cloth via a hydrothermal synthesis and subsequent annealing treatment.^153^ The electrode showed good cycling performance with a capacity of 892.6 mAh g^–1^ at 100 mA g^–1^ after 120 cycles and a capacity of 758.1 mAh g^–1^ at 700 mA g^–1^ after 150 cycles. A high rate capability was achieved with a capacity of 298.4 mAh g^–1^ at high current density of 5000 mA g^–1^. The durable high rate performance could be ascribed to the unique mesoporous structure that is beneficial to the mass transport of electrolytes and the diffusion of Li^+^ ions.

Considerable attention has been paid to the fabrication of hierarchical nanoarrays of spinel ternary metal oxides, such as ZnCo_2_O_4_,^154^ NiCo_2_O_4_,^155^ MnCo_2_O_4_,^156^ and Co_x_Mn_3–x_O_4_,^157^ on 3D conductive substrates. For example, urchin‐like ZnCo_2_O_4_ nanoarrays were fabricated on carbon cloth by a simple hydrothermal process.^158^ The urchin‐like ZnCo_2_O_4_ nanostructures are composed of radially aligned porous nanowires. When tested as an anode in LIBs, a capacity of 1180 mAh g^–1^ could be retained at 0.2 C (1 C = 900 mA g^–1^) after 100 cycles. Capacities of 900 and 750 mAh g^–1^ were achieved after 100 cycles at 5 C and 20 C, respectively. As another example, leaf‐like, porous ZnCo_2_O_4_ nanowire arrays were grown on Ni foam through a hydrothermal process followed by a post annealing treatment.^159^ The leaf‐like ZnCo_2_O_4_ nanowire array electrode exhibited a good cycling performance with a capacity of 1050 mAh g^–1^ at 100 mA g^–1^ after 60 cycles. A capacity of 240 mAh g^–1^ was retained at a high rate of 2778 mA g^–1^.

**Figure 12:**
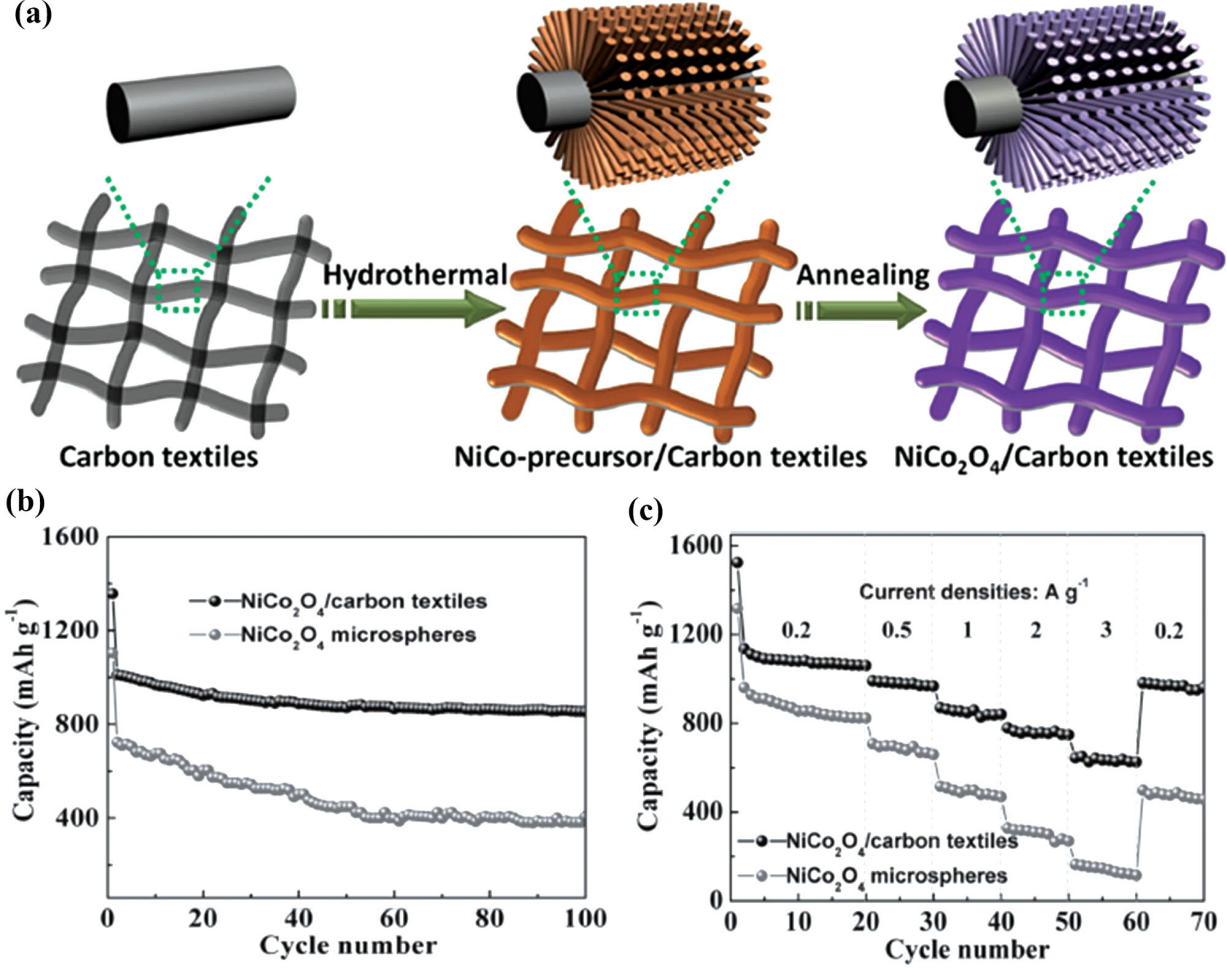
a) Schematic illustration of the formation of NiCo_2_O_4_/carbon textiles. b) Cycling performance at 500 mA g^–1^ and c) rate capability of NiCo_2_O_4_/carbon textiles and NiCo_2_O_4_ microspheres. Reproduced with permission.^160^

Moreover, mesoporous NiCo_2_O_4_ nanowire arrays were produced on carbon textiles by a simple surfactant‐assisted hydrothermal method combined with a post annealing treatment.^160^ As shown in **Figure** **12**a, the NiCo‐precursor nanowire arrays were firstly fabricated on carbon textiles under hydrothermal condition, and then the conversion into spinel NiCo_2_O_4_ nanowire arrays were realized via annealing. The NiCo_2_O_4_ nanowires composed of small nanoparticles had a mesoporous structure with an average pore size around 6 nm. When investigated as an anode in LIBs, a high discharge capacity of 854 mAh g^–1^ was achieved at 500 mA g^–1^ for NiCo_2_O_4_ nanowire arrays after 100 cycles, which was superior to that of NiCo_2_O_4_ microspheres (≈400 mAh g^–1^), as shown in Figure 12b. Significantly improved rate capability was also observed for the NiCo_2_O_4_ nanowire arrays (see Figure 12c). The preferable electrochemical performance of NiCo_2_O_4_ nanowire arrays could be attributed to the robust adhesion between NiCo_2_O_4_ nanowire arrays and carbon textiles that ensures the effective electron transport, the loose textiles and large open spaces allowing electrolyte to easily contact with NiCo_2_O_4_, and the mesoporous structure that accommodates the volume changes and shortens the ion‐diffusion length. Recently, 3D porous NiCo_2_O_4_ nanowire arrays were fabricated on carbon cloth via a facile low‐cost solution method combined with a subsequent annealing process.^161^ The NiCo_2_O_4_ nanowire array electrode exhibited a good cycling performance with a capacity of 1085.5 mAh g^–1^ at 500 mA g^–1^ after 100 cycles. A capacity of 507 mAh g^–1^ was retained at a high rate of 4000 mA g^–1^.

Furthermore, hierarchical MnCo_2_O_4_ nanosheet arrays grown on carbon cloth exhibited good cycling performance with an areal capacity of 3 mAh cm^–2^ at 800 μA cm^–2^ after 60 cycles, as well as a good rate capability with a capacity of 2 mAh cm^–2^ retained at 1600 μA cm^–2^.^162^ Additionally, porous FeCo_2_O_4_ nanoneedle arrays grown on Ni foam demonstrated a capacity of 1335 mAh g^–1^ at 100 mA g^–1^ after 200 cycles, and showed a good rate capability with a capacity of 875 mAh g^–1^ retained at 2000 mA g^–1^.^163^

### 4.3 Heterostructured Nanoarrays

As mentioned above, heterostructured nanoarrays composed of composite metal oxides may bring about complementary and/or synergistic effects and hence represent a promising candidate for high‐performance nanoarray electrodes for LIBs. In this regard, considerable effort has been devoted to the fabrication of heterostructured metal oxide nanoarrays on 3D porous substrates as binder‐free electrodes for advanced LIBs.^164^, ^165^, ^166^ For example, hierarchically porous TiO_2_ nanotube@SnO_2_ nanoflake core‐branch nanoarrays were fabricated on Ni foam through atomic layer deposition (ALD) followed by hydrothermal growth.^167^ The Co(OH)_2_CO_3_ nanorod arrays were first employed as template for the atomic layer deposition of TiO_2_ nanotubes; after template removal, SnO_2_ nanoflakes were grown on the robust TiO_2_ nanotube stems by hydrothermal growth. In this 1D core‐branch nanostructure, the TiO_2_ nanotube stem offered a low‐mass scaffold for the SnO_2_ nanoflakes and also a charge conductive path, leading to enhanced lithium storage properties. When evaluated as an anode in LIBs, the obtained TiO_2_ nanotube@SnO_2_ nanoflake nanoarray electrode showed improved cycling and rate performance with a capacity of around 580 mAh g^–1^ at 1.6 A g^–1^ after 50 cycles and a capacity of 498 mAh g^–1^ retained at a high rate of 3.2 A g^–1^. On the other hand, a novel wire‐in‐tube nanostructure of SnO_2_‐in‐TiO_2_ nanoarrays were fabricated on carbon cloth through vapor deposition followed by ALD.^168^ The gap between SnO_2_ nanowire and TiO_2_ shell allowed a nearly free expansion of SnO_2_ nanowire without severe pulverization. The SnO_2_‐in‐TiO_2_ nanoarray electrode showed excellent cycling performance with stable capacities of 494.9 and 393.3 mAh g^–1^ at 400 mA g^–1^ after 500 and 1000 cycles, respectively. A high rate capability was achieved with a capacity of 241.2 mAh g^–1^ at 3200 mA g^–1^.

Owing to the high theoretical capacity, nontoxicity, abundance, and easy preparation, Co_3_O_4_ nanorod arrays grown on Ni foam have been frequently used for the deposition of nanostructures of another metal oxide, such as Fe_2_O_3_,^169^ NiO,^170^ and MnO_2_,^171^ yielding heterostructured nanoarray electrodes with enhanced electrochemical performance. Specifically, the produced Co_3_O_4_/MnO_2_ nanoarrays on Ni foam exhibited high reversible capacity of 1060 mAh g^–1^ at a rate of 120 mA g^–1^ together with good cycling stability and rate capability when evaluated as an anode for LIBs; meanwhile, they showed high specific capacitance, long‐term cycling stability, and high energy density when used for supercapacitors.^171^

Recently, 1D CuO nanoarrays grown on 3D porous substrates have been employed as new backbones for the fabrication of heterostructured nanoarrays based on CuO 1D nanostructure cores. Specifically, Zhang and co‐workers reported the preparation of hierarchical CuO/Co_3_O_4_ core‐shell nanowire arrays on Ni foam.^172^ The CuO nanowire arrays were first fabricated on Ni foam via a facile thermal oxidation method. Then the branched Co_3_O_4_ nanosheets were grown on the surface of the CuO nanowire arrays via a chemical bath deposition (CBD) followed by a calcination process. The Co_3_O_4_ nanosheets were highly porous and consisted of many interconnected small nanocrystals with a diameter of 5–15 nm. When investigated as an anode in LIBs, the CuO/Co_3_O_4_ heterostructure nanoarrays demonstrated excellent cyclability with stable specific capacities of 1191 mAh g^–1^ and 810 mAh g^–1^ at 200 mA g^–1^ and 1000 mA g^–1^ after 200 cycles, respectively. Even at a high current density of 2500 mA g^–1^, a charge capacity of 580 mAh g^–1^ was delivered, indicating improved rate capability. The excellent electrochemical performance of the heterostructured arrays originated from the synergistic effect of CuO and Co_3_O_4_ in terms of the good mechanical adhesion, fast charge transfer pathways, and the shortened Li^+^ diffusion lengths.

**Figure 13:**
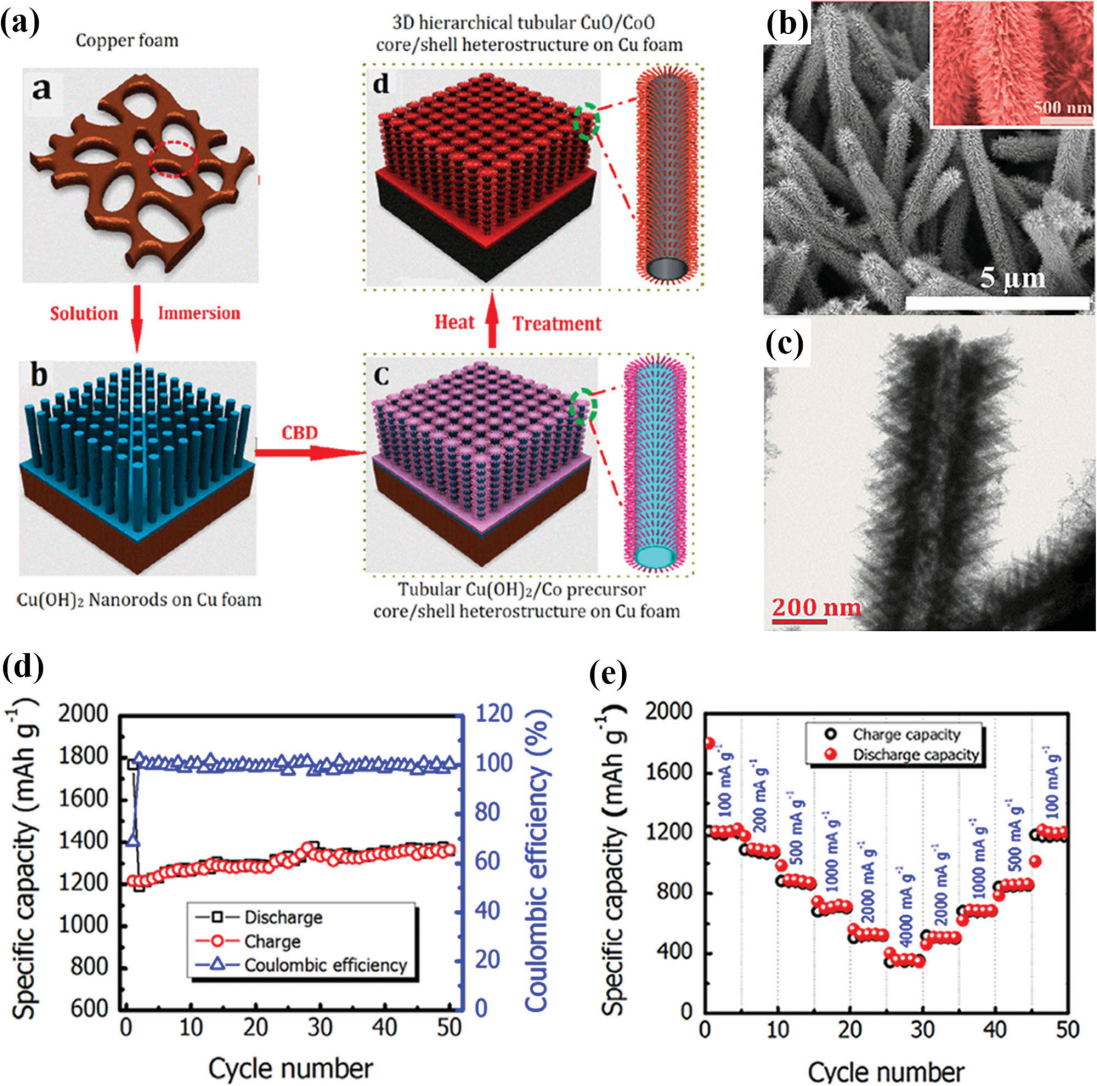
a) Schematic illustration for the fabrication process of 3D hierarchical tubular CuO/CoO core/shell heterostructure arrays on Cu foam; b) SEM and c) TEM images of the hierarchical tubular CuO/CoO core/shell heterostructure. d) Cycling performance of 3D hierarchical tubular CuO/CoO core/shell heterostructure arrays on Cu foam at 100 mA g^–1^. e) Rate capability of the heterostructure arrays in the current range of 100–4000 mA g^–1^. Reproduced with permission.^173^ Copyright 2015, Elsevier.

Later on, Zhang and co‐workers realized the fabrication of hierarchical tubular CuO/CoO core‐shell heterostructure arrays on Cu foam.^173^ The fabrication process of the 3D hierarchical tubular CuO/CoO heterostructure arrays is illustrated in **Figure** **13**a. Firstly, high density Cu(OH)_2_ nanorod arrays were prepared on copper foam as backbones through a solution immersion method. Then, cobalt precursor nanosheets were fabricated uniformly on the Cu(OH)_2_ nanorod backbones by a CBD method. Finally, the as‐obtained 3D hierarchical tubular Cu(OH)_2_/Co precursor heterostructure arrays were converted to 3D hierarchical tubular CuO/CoO core/shell heterostructure arrays through calcination. The SEM image shown in Figure 13b reveals that the CoO branches were successfully coated onto the CuO nanotube core with a good uniformity and homogenous distribution along the nanorod. The TEM image shown in Figure 13c reveals the hollow interior of the CuO nanotubes with numerous tiny CoO nansheets attaching to the surface. When evaluated as a binder‐free anode for LIBs, these 3D hierarchical tubular CuO/CoO core/shell heterostructure arrays exhibited excellent cycling performance with an impressive capacity of 1364 mAh g^–1^ after 50 cycles at 100 mA g^–1^ (Figure 13d). A capacity of 342 mAh g^–1^ was maintained at a high rate of 4000 mA g^–1^ (Figure 13e), indicating a high rate capability. The superior electrochemical performance could be attributed to the synergistic effects of the hollow CuO nanotube backbone combined with branched CoO nanosheet shells: First, the highly ordered 1D geometry of the tubular CuO backbone was electrically connected to the 3D porous Cu foam that reduced the resistance for electron injection from the current collector to the electrode, leading to fast charge/discharge capabilities. Second, the mesoporous CoO nanosheets facilitated electrolyte penetration into the inner regions of the CuO nanotube cores as well as the rest of the composite, enabling a high Li^+^ ion flux across the interface, which accounted for the high reversible capacity and good rate capability. Thirdly, the hollow CuO cores were connected to Cu foam with strong adhesion and were able to maintain mechanical integrity, thereby enhancing the cycle stability.

## 5 Applications of Self‐Supported Metal Oxide Nanoarrays in Full Cells

The most common technique in laboratory to evaluate a self‐supported nanoarray electrode is to utilize the tested sample as the working electrode (cathode) and a lithium foil as the counter electrode (anode) to assemble into a half‐cell with the addition of organic electrolyte. Under these circumstances, the tested materials are always tested as the cathode in “half cells”. Although lithium metal has been regarded as the most ideal anode in LIBs owing to its highest energy densities with sustaining supply of excess and sufficient Li^+^ ions, the trade‐offs in turn are triggered such as the safety issues associated with the forming of dendrite structures with high surface areas. In this respect, the realization of the transition from half‐cells to full‐cell configurations plays a crucial role for the practical applications of the self‐supported metal oxide nanoarray electrodes. However, some obstacles remain to be resolved when the self‐supported nanoarray electrodes are assembled into full cells: First, the relatively large disparities between the specific capacities of cathode and anode materials usually result in the different mass ratios of the active materials in the electrodes. Considering the large irreversible capacity loss due to the formation of solid electrolyte interphase (SEI) layer during the initial cycles for metal oxides, the determination of the exact mass ratios of the two electrodes is difficult, leading to the unnecessary waste of partial active materials. Second, when tested as electrodes in full cells, the difference in the lithiation/delithiation mechanism between cathode intercalation materials and anode conversion materials probably leads to declined specific capacities and poor rate capabilities in comparison to the half cell tests. Third, unlike the lithium metal electrodes, cathode materials such as LiCoO_2_, LiMn_2_O_4_ and LiFePO_4_, have limited capabilities of offering sufficient Li^+^ ions. Thus undesirable lithium loss will occur due to the potential side reactions between metal oxides and electrolyte, leading to a poor cycling performance resulting from the unexpected capacity loss. Lastly, compared with the conventional thin film batteries, the array‐based batteries still suffer from the immature package techniques in terms of air tightness and safety. Therefore, the rational design and construction of desirable full cells from self‐supported metal oxide nanoarray electrodes are receiving increasing attention.^174^, ^175^, ^176^, ^177^

**Figure 14:**
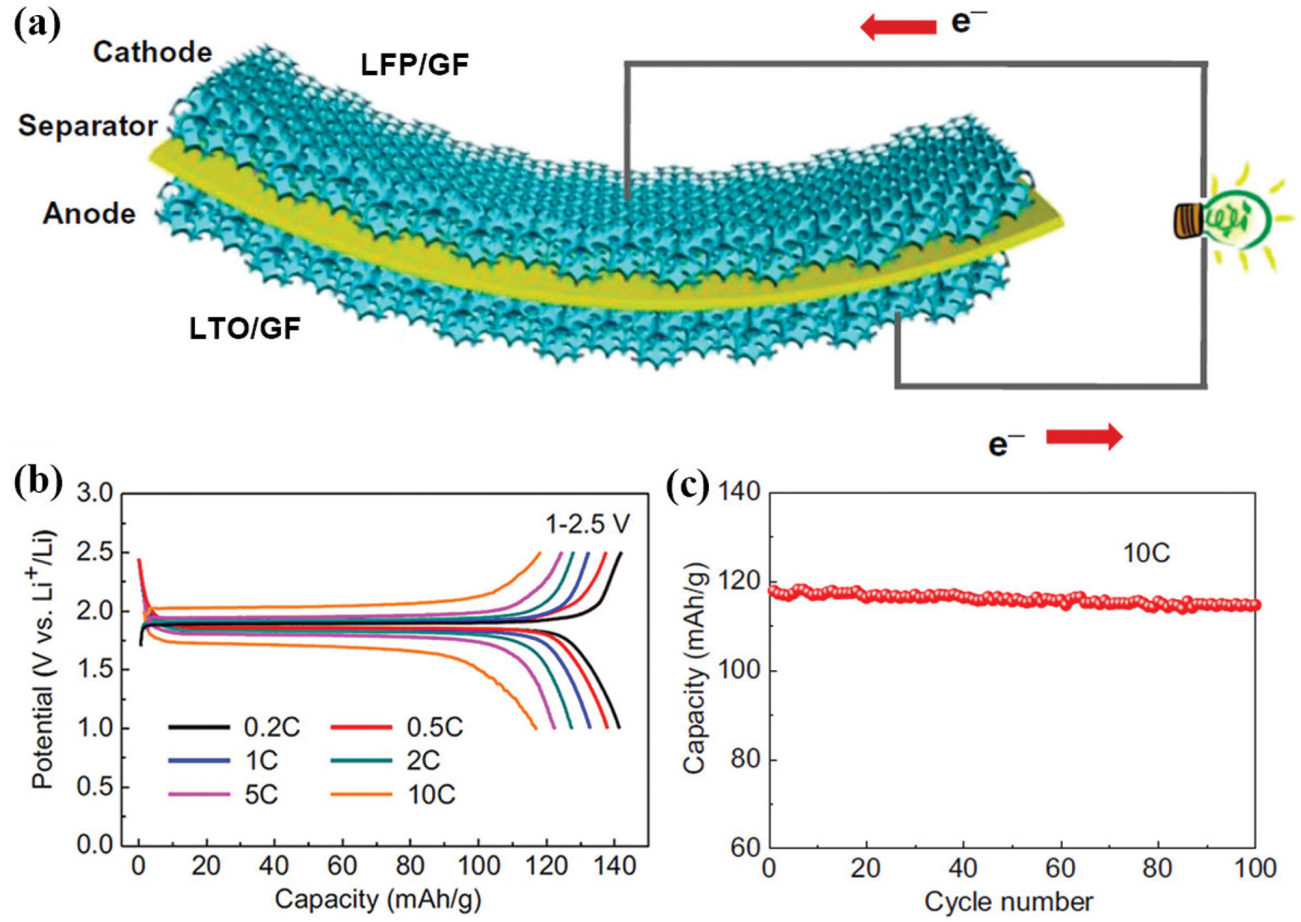
a) Schematic of a flexible battery containing a cathode LiFePO_4_ (LFP) and an anode Li_4_Ti_5_O_12_ (LTO) made from 3D interconnected GF. b) Charging/discharging voltage curves of LTO/GF//LFP/GF full battery with different current rates in the voltage range of 1.0–2.5 V. c) Cyclic performance of a flexible LTO/GF//LFP/GF full battery at a constant 10C rate for 100 cycles. Reproduced with permission.^132^ Copyright 2012, National Academy of Sciences.

As described above, the Li_4_Ti_5_O_12_ nanosheet arrays grown on 3D graphene foam (LTO/GF) exhibited outstanding cycling and rate performance in LIB half‐cell tests.^132^ When the LTO/GF anode was combined with a cathode with LiFePO_4_ nanoparticles on graphene foam (LFP/GF), a flexible LTO/GF//LFP/GF full battery with high rate performance and energy density were realized (**Figure** **14**a). As shown in Figure 14b, a capacity of 117 mAh g^–1^ at 10 C (1 C = 145 mA g^–1^, based on the mass of cathodic LFP) in the voltage window of 1.0–2.5 V was achieved for the full cell. Figure 14c shows that 96% of the initial capacity could be retained at 10 C after 100 cycles, indicating excellent cycling performance for the full battery. Recently, 3D web‐like structures comprising Li_4_Ti_5_O_12_ nanowires on Ti foil were fabricated by a facile hydrothermal method and used a binder‐free anode for flexible LIBs.^178^ A flexible full battery assembled from the web‐like LTO nanostructures on Ti foil as the anode and LiMn_2_O_4_ (LMO) nanorods coated on stainless steel cloth as the cathode exhibited a capacity of 168 mAh g^–1^ after 70 cycles at current rate of 2 C (1 C = 175 mA g^–1^, based on the mass of anodic LTO). A capacity of 120 mAh g^–1^ after 70 cycles at a high rate of 20 C was achieved, indicating excellent rate performance.

**Figure 15:**
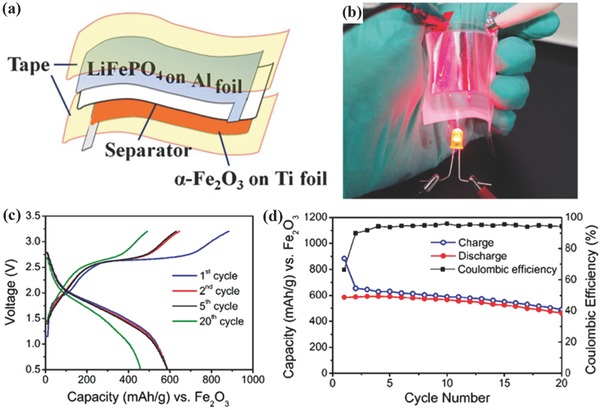
a) Schematic illustration of a lithium ion full cell assembled using α‐Fe_2_O_3_ nanorod arrays with optimized interstices (Fe_2_O_3_‐NA‐1.5) on Ti foil as the anode. b) Optical photograph of an LED lightened by the bended battery. c) Voltage profiles and d) cycling performance of the flexible battery at 5 C. Reproduced with permission.^98^ Copyright 2015, Royal Society of Chemistry.

In addition to the Li_4_Ti_5_O_12_ nanoarray electrodes, hierarchical α‐Fe_2_O_3_ nanoarray electrodes have also been assembled into full lithium ion batteries. As mentioned above, the etched mesocrystalline α‐Fe_2_O_3_ nanorod arrays with optimized interspaces (Fe_2_O_3_‐NA‐1.5) on Ti foil demonstrated remarkable electrochemical performance when evaluated as anode in LIBs.^98^ For full cell tests, a flexible full lithium ion battery was assembled using Fe_2_O_3_‐NA‐1.5 on Ti foil as the anode and commercial LiFePO_4_ powders loaded on Al foil as the cathode (**Figure** **15**a,b). The α‐Fe_2_O_3_ nanoarray electrode exhibited initial charge and discharge capacities of 884 and 586 mAh g^–1^, respectively, and retained a stable reversible capacity within 20 cycles (Figure 15c,d). However, the specific capacity and cyclability of the nanoarray electrode in full batteries were somewhat lower than those in half batteries, which might be attributed to the increasing internal resistance and the sealing deficiency. As another example, hierarchical porous α‐Fe_2_O_3_ nanosheet arrays grown on Cu foil showed excellent cycling stability and rate capability in half cell tests.^100^ When a full battery was assembled from the α‐Fe_2_O_3_ nanoarray anode and a LiFePO_4_ cathode, a capacity of 435 mAh g^–1^ was maintained at 0.5 C (1 C = 1006 mA g^–1^, with reference to the anodic α‐Fe_2_O_3_ mass) after 30 cycles, which corresponded to only 52.7% of the initial discharge capacity. Obviously, the electrochemical performance obtained from full cell tests was inferior to that from half cell tests, which might be related to the mismatched current densities of the two electrodes, an unreasonable mass ratio of the electrodes, and the selection of inappropriate electrolyte.

Furthermore, a full battery consisting of CuO nanorod array anode versus high voltage spinel LiNi_0.5_Mn_1.5_O_4_ cathode was produced and implemented in full cell tests.^179^ The CuO/Li half cell test of the CuO nanorod arrays on Cu substrate showed a stable discharge capacity of 666 mAh g^–1^ with capacity retention of 91% at 0.5 C (1 C = 674 mA g^–1^) after 100 cycles, and a relatively high capacity of 560 mAh g^–1^ at a high rate of 10 C. In contrast to the half cell tests, the results from full cell tests showed a discharge capacity of 516 mAh g^–1^ with a capacity retention of 84% at 0.5 C (1 C = 674 mA g^–1^, based on the mass of anodic CuO) after 100 cycles. Impressively, the CuO/LiNi_0.5_Mn_1.5_O_4_ full cell demonstrated superior rate capability with a reversible capacity of 240 mAh g^–1^ at a high rate of 10 C. The good cyclability and excellent rate capability could be ascribed to the unique structures of the CuO nanorod arrays and the hierarchical LiNi_0.5_Mn_1.5_O_4_ microspheres.

In addition, ternary metal oxides have also been applied in full‐cell tests.^162^, ^180^ As mentioned above, the electrode made of MnCo_2_O_4_ nanosheet arrays grown on carbon cloth showed good half‐cell performance.^162^ When assembled into full cells with the commercial LiCoO_2_/Al as the cathode, the MnCo_2_O_4_ nanosheet array electrode demostrated improved rate capability compared with Co_3_O_4_‐based full cell. Besides, CoMoO_4_/polypyrrole core–shell nanowire arrays were successfully constructed on carbon cloth, which were used as a binder‐free anode to assemble full cells using commercial LiFePO_4_ on Al foil as a cathode.^180^ The full‐cell battery exhibited an excellent cycling performance with a capacity of about 400 mAh g^–1^ at 400 mA g^–1^ after 1000 cycles in the voltage range of 2.0–3.8V. A capacity of 80 mAh g^–1^ was retained at a high rate of 1500 mA g^–1^.

It may be noted that self‐supported metal oxide nanoarrays grown on both 2D planar and 3D porous substrates have been employed as binder‐free electrodes for constructing full‐cell batteries, especially for flexible full batteries. In general, the electrochemical performance of the 2D nanoarray electrodes is less affected by the packing techniques because of their similarity with commercial planar electrodes, but the areal capacity is usually low owing to the low loading mass of the active materials. Although the high porosity and loading mass of 3D nanoarray electrodes lead to enhanced capacity and rate capability in half‐cell test, their electrochemical performance is strongly influenced by the fabrication conditions of the full‐cell batteries. It remains a challenge to realize full‐cell performance comparable to the performance obtained in half‐cell tests for the 3D nanoarray electrodes.

**Figure 16:**
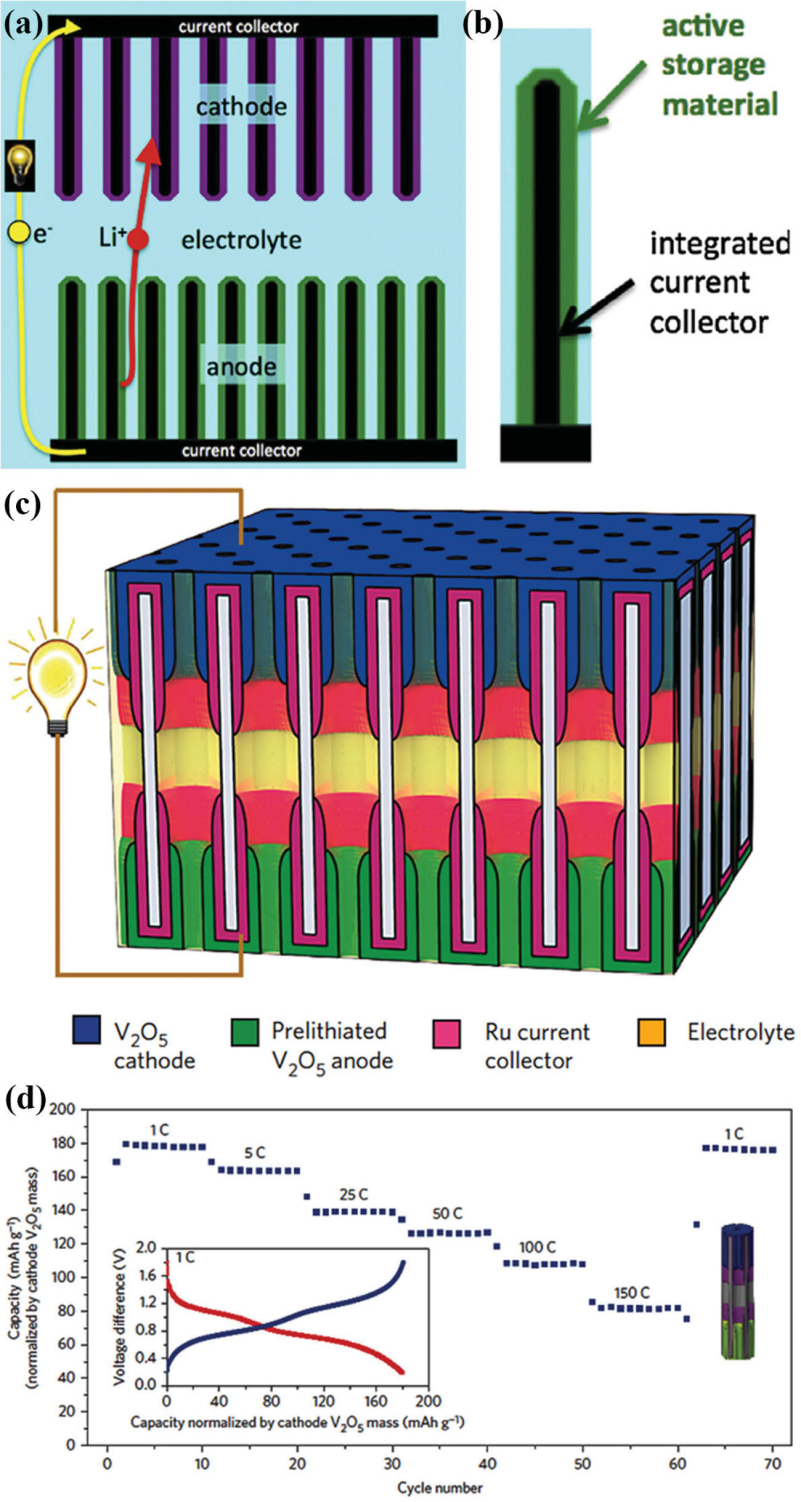
a) Schematic illustration of heterogeneous nanowire electrodes. b) Integrated ion storage and electron current collecting materials comprise heterogeneous nanoelectrodes. Reproduced with permission.^181^ Copyright 2015, Elsevier. c) Schematic of parallel nanopore battery array and cross‐section of a single‐pore battery. d) Rate performance of a symmetric full‐cell device (capacity normalized by cathode V_2_O_5_ mass). Inset: Charge and discharge curves at 1 C. Reproduced with permission.^186^ Copyright 2014, Macmillan Publishers Limited.

Despite considerable efforts devoted to full cell tests, few of them can take advantage of both anode and cathode made of metal oxide nanoarrays in an integrated full cell simultaneously. One reason lies in the difficulties in the fabrication of metal oxide nanoarray cathodes. Another reason could be related to the uneven electron transport lengths from different redox active sites to the bottom of current collector, which may give rise to unequal electrochemical reaction kinetics.^126^ Furthermore, the low loading mass of active materials for metal oxide nanoarrays on 2D planar substrate results in low specific areal capacity and energy density, limiting their practical utilization. To solve these problems, depositing active materials on 3D metal nanoarray current collector with reduced internal impedance, uniform electron transport length from active materials to current collector, and high loading mass of active materials provides an exciting platform for achieving high energy density and power density (**Figure** **16**a,b),^181^ which has received considerable attention and shown special significance for 3D lithium‐ion microbatteries.^182^, ^183^, ^184^, ^185^ However, a main drawback of these 3D metal nanoarray electrodes in full cell configurations is that the confined electrolyte environments result in insufficient Li^+^ ion supply and the ion diffusion from bulk electrolyte is too slow to satisfy high‐rate capability. Impressively, Liu et al.^186^ reported a full battery comprising Ru‐nanotube current collectors with V_2_O_5_ storage material confined within anodic aluminum oxide (AAO) nanopores, forming a symmetric cell where the LiV_2_O_5_ anode and the pristine V_2_O_5_ cathode were separated by an electrolyte region, as shown in Figure 16c. When tested in full cell device, this “all‐in‐one” nanopore battery had a capacity of 150 mAh g^–1^ at 1 C and 70 mAh g^–1^ at 150 C (1 C = 147mA g^–1^, based on the mass of cathodic V_2_O_5_) with a voltage window from –1 V–1 V. As the voltage window was increased to 0.8 V–1.8 V, the specific capacity of 178 mAh g^–1^ at 1 C and 82 mAh g^–1^ at 150 C (normalized by the mass of the anode) was comparable to that of the half cell (Figure 16d). The results indicate that the optimized nanoelectrode geometries can avoid the disadvantages related to the extreme confinement of the electrolyte solution.

## 6 Conclusions and Prospects

Although LIBs are currently the most widely used electrochemical energy storage devices, their development has largely been limited by some bottlenecks such as low energy density, low power density, and poor cycle stability. As one of the effective solutions to these problems, the utilization of nanostructured metal oxides with high specific capacity as high‐performance electrodes for LIBs has received considerable attentions. However, the existing drawbacks including the large irreversible capacity loss during initial cycles, huge volume expansion during lithiation and poor charge transfer kinetics greatly handicap their applications in LIBs. As a new class of potential alternatives for replacing commercial electrodes, the self‐supported metal oxide nanoarray electrodes have been extensively investigated owing to their numerous merits such as high specific surface area, shortened transport length for lithium ion diffusion, direct pathways for electron transfer, good contact between nanoarrays and current collector, facile strain relaxation for large volume expansion, and capability to act as binder‐free electrodes.^19^, ^187^ In this regard, plenty of self‐supported metal oxide nanoarrays with a variety of morphologies grown directly on 2D planar/3D porous substrates have flourished. Specifically, a number of 1D metal oxide nanostructures, including nanowires, nanorods, nanobelts, and nanotubes, and a variety of 2D metal oxide nanostructures including nanosheets, nanoplates, nanoflakes, and nanowalls have been successfully fabricated on 2D/3D conductive substrates as binder‐free electrodes with enhanced cyclability and rate capability. Moreover, hierarchical metal oxide nanoarrays comprising nanoscale subunits are generally characterized by a hierarchical porous structure. The multiple pores including micropores, mesopores, and macropores are accessible for the penetration of electrolyte, facilitating the charge transfer rate and improving electrochemical reaction kinetics. Furthermore, heterostructured nanoarrays composed of composite metal oxides have shown great potential in generating hybrid nanoarray electrodes with enhanced capacity, cyclability, and rate capability because of the complementary and/or synergistic effects. Generally, the fabrication of self‐supported metal oxide nanoarrays on conductive substrates can be classified into template‐free methods and template methods. The template‐free methods, such as hydrothermal/solvothermal synthesis, electrochemical anodization, and vapor phase deposition, usually involve simple fabrication processes but are applicable to limited materials. The template methods can be applied to a broader range of materials but they require prefabricated templates with desirable 1D or 2D structures, which should be easy to be fabricated, able to act as templates, and easy to be removed.

2D planar substrates, such as commercial copper foil, titanium foil, and stainless steel foil, hold the virtues of good thermal and chemical stability, high electronic conductivity, and simple fabrication techniques, providing a commodious platform for the growth of various metal oxide nanoarrays. **Table** **1** summarizes the cycling performance and rate capability of different metal oxide nanoarrays grown on 2D planar substrates mentioned in this review. Such metal oxide nanoarrays on 2D planar substrates with high charge transfer efficiency often give rise to improved electrochemical performance. Moreover, the lack of binders and additives largely reduced the whole electrode mass and the internal interfacial resistance, leading to high specific gravimetric capacity and increased electron transport ability. However, the low loading mass of active materials due to the limited growth sites normally results in low areal capacity and whole energy of the electrode. In contrast, 3D porous substrates, such as commercial nickel foam, copper foam, graphene foam, and carbon cloth, are able to afford enhanced loading mass of active materials, which is greatly beneficial for the improvement of the areal capacity and the whole energy of the electrode. **Table** **2** summarizes the cycling performance and rate capability of different metal oxide nanoarrays grown on 3D porous substrates mentioned in this review. Particularly, the existence of macropores inside the electrode has great significance for the fast infiltration of electrolyte. As a consequence, such 3D porous electrodes often have conspicuous high rate performance due to the intensive electrochemical reaction kinetics and enhanced charge diffusion efficiency. Nevertheless, relatively low specific volumetric capacity and tap density are often observed in 3D porous electrodes due to the large void spaces inside the electrode. It is noteworthy that the loading mass of active materials in nanoarray electrodes depends not only on the geometry of current collector but also the thickness of the growing materials. A thin layer of active materials tend to exhibit better electrochemical performance, especially for rate capability, which is yet not easily to be achieved with a thicker layer of the active materials even using 3D porous substrates. It remains a challenge to achieve high battery performance for nanoarray electrodes containing a thick layer of active materials through rational design of electrode architectures. Furthermore, the gravimetric capacity of the whole electrode, which consists of active materials and substrates, is usually low for the nanoarray electrodes with a large mass of the substrate. Possible solutions to this problem include the increase of the thickness of the growth layer coupled with electrode design and the decrease of the mass of the substrate by selecting light‐weight substrates.

**Table 1:** Cycling stability and rate capability of different metal oxide nanoarrays grown on 2D planar substrates

Nanostructures	2D substrate	Cycling stability (after *n* cycles)	Rate capability	Loading mass of active materials (mg cm^–2^)	Reference
SnO_2_ nanorods	Fe‐based alloy substrate	78.1 mA g^–1^, 580 mAh g^–1^, *n* = 100	3905 mA g^–1^, 350 mAh g^–1^	0.31	44
SnO_2_ nanowires	Stainless steel foil	782 mA g^–1^, 510 mAh g^–1^, *n* = 50	7820 mA g^–1^, 440 mAh g^–1^		45
Carbon‐coated SnO_2_ nanorods	Fe‐Co‐Ni alloy substrate	500 mA g^–1^, 585 mAh g^–1^, *n* = 50	3000 mA g^–1^, 320 mAh g^–1^		46
SnO_2_ nanosheets	Titanium foil	200 mA g^–1^, 260 μAh cm^–2^, *n* = 30	1500 mA g^–1^, 400 mAh g^–1^	0.8	78
SnO_2_ nanorods	Titanium foil	3910 mA g^–1^, 780 mAh g^–1^, *n* = 20	7820 mA g^–1^, 590 mAh g^–1^	0.55	86
Carbon/ZnO nanorods	Nickel foil	247 mA g^–1^, 330 mAh g^–1^, *n* = 50	741 mA g^–1^, 360 mAh g^–1^	0.7	50
ZnO microrods	Copper foil	500 mA g^–1^, over 500 mAh g^–1^, *n* = 100	2000 mA g^–1^, 220 mAh g^–1^		51
CuO nanoribbons	Copper foil	175 mA g^–1^, 608 mAh g^–1^, *n* = 275	800 mA g^–1^, 332 mAh g^–1^	0.4	55
CuO nanorods	Copper foil	325 mA g^–1^, 650 mAh g^–1^, *n* = 100	1300 mA g^–1^, 450 mAh g^–1^		56
CuO nanosheets	Copper foil	674 mA g^–1^, 639.8 mAh g^–1^, *n* = 100	6740 mA g^–1^, 548.8 mAh g^–1^		102
Li_4_Ti_5_O_12_‐C nanotubes	Stainless steel foil	1750 mA g^–1^, 150 mAh g^–1^, *n* = 500	17500 mA g^–1^, 80 mAh g^–1^	0.42	64
Li_4_Ti_5_O_12_ nanosheets	Titanium foil	8750 mA g^–1^, 124 mAh g^–1^, *n* = 3000	35000 mA g^–1^, 78 mAh g^–1^	0.04	73
Anatase TiO_2_ nanotubes	Titanium foil	0.05 mA cm^–2^, 0.46 mAh cm^–2^, *n* = 100	2.5 mA cm^–2^, 0.24 mAh cm^–2^	2.5	71
LiMn_2_O_4_ nanorods	Pt foil	148 mA g^–1^, 113 mAh g^–1^, *n* = 200	1480 mA g^–1^, 106 mAh g^–1^		82
LiCoO_2_ nanowires	Au substrate	14.8 mA g^–1^, ≈120 mAh g^–1^, *n* = 50	1480 mA g^–1^, 102 mAh g^–1^	0.3–0.5	108
V_2_O_5_ nanobelts	Titanium foil	50 mA g^–1^, 255 mAh g^–1^, *n* = 50			81
NiO nanowalls	Nickel foil	179 mA g^–1^, 638 mAh g^–1^, *n* = 85	1339 mA g^–1^, 490 mAh g^–1^	0.17	75
Co_3_O_4_ nanowires	Titanium foil	111 mA g^–1^, 780 mAh g^–1^, *n* = 20	5550 mA g^–1^, 240 mAh g^–1^	2–3	88
Co_3_O_4_ nanobelts	Titanium foil	67 mA g^–1^, 770 mAh g^–1^, *n* = 25	3330 mA g^–1^, 330 mAh g^–1^	0.6–0.9	89
CoO nanowires	Titanium foil	716 mA g^–1^, 670 mAh g^–1^, *n* = 20	4296 mA g^–1^, 150 mAh g^–1^		94
SiO_2_‐coated CoO nanowires	Copper foil	716 mA g^–1^, ≈1200 mAh g^–1^, *n* = 200	3580 mA g^–1^, ≈1000 mAh g^–1^	0.95	95
α‐Fe_2_O_3_ nanorods	Titanium foil	134.2 mA g^–1^, 562 mAh g^–1^, *n* = 50	1342 mA g^–1^, 459 mAh g^–1^		97
α‐Fe_2_O_3_ nanorods	Titanium foil	5000 mA g^–1^, 970 mAh g^–1^, *n* = 500	30000 mA g^–1^, 350 mAh g^–1^	0.042	98
Porous α‐Fe_2_O_3_ nanosheets	Copper foil	2012 mA g^–1^, 850 mAh g^–1^, *n* = 1000	20120 mA g^–1^, 433.2 mAh g^–1^	0.35	100
Porous α‐Fe_2_O_3_ nanosheets	Titanium foil	100 mA g^–1^, 908 mAh g^–1^, *n* = 60	2000 mA g^–1^, 573 mAh g^–1^	0.38	101
NiCo_2_O_4_ nanorod arrays	Copper foil	430 mA g^–1^, 830 mAh g^–1^, *n* = 30	94600 mA g^–1^, 127 mAh g^–1^		103
CoMn_2_O_4_ nanowire arrays	Stainless steel foil	800 mA g^–1^, 450 mAh g^–1^, *n* = 30	7000 mA g^–1^, 215 mAh g^–1^	0.38	104
MnCo_2_O_4_ nanosheet arrays	Stainless steel foil	800 mA g^–1^, 460 mAh g^–1^, *n* = 30	7000 mA g^–1^, 270 mAh g^–1^	0.38	104
Cu_x_Co_3–x_O_4_ nanosheet arrays	Titanium foil	400 mA g^–1^, 1107 mAh g^–1^, *n* = 50	55000 mA g^–1^, 335 mAh g^–1^		105
TiO_2_‐C/MnO_2_ nanowires	Titanium foil	33.5 mA g^–1^, 352 mAh g^–1^, *n* = 100	10050 mA g^–1^, 130 mAh g^–1^	1.5	117
Li_4_Ti_5_O_12_‐TiO_2_ nanowires	Titanium foil	1750 mA g^–1^, 129.3 mAh g^–1^, *n* = 100	5250 mA g^–1^, 115.5 mAh g^–1^		118
SnO_2_/α‐Fe_2_O_3_ nanotubes	Stainless steel foil	0.1 mA cm^–2^, 0.727 mAh cm^–2^, *n* = 50	0.6 mA cm^–2^, 0.507 mAh cm^–2^	0.75	123

**Table 2:** Cycling stability and rate capability of different metal oxide nanoarrays grown on 3D porous substrates

Nanostructures	3D substrate	Cycling stability (after *n* cycles)	Rate capability	Loading mass of active materials (mg cm^–2^)	Reference
SnO_2_ nanorods	Nickel foam	156 mA g^–1^, 607 mAh g^–1^, *n* = 50			129
SnO_2_‐PANI nanorods	Nickel foam	200 mA g^–1^, 506 mAh g^–1^, *n* = 100	3000 mA g^–1^, 312 mAh g^–1^	1.67	130
SnO_2_‐PPy nanorods	Nickel foam	200 mA g^–1^, 701 mAh g^–1^, *n* = 300	3000 mA g^–1^, 512 mAh g^–1^	1.67 ± 0.35	131
Li_4_Ti_5_O_12_ nanosheets	Graphene foam	5250 mA g^–1^, 150 mAh g^–1^, *n* = 500	35000 mA g^–1^, 86 mAh g^–1^	0.83	132
α‐Fe_2_O_3_ nanowalls	Nickel foam	100.7 mA g^–1^, 518 mAh g^–1^, *n* = 50	5035 mA g^–1^, 440 mAh g^–1^		133
MnO_2_ nanoflakes	Graphene foam	500 mA g^–1^, 1200 mAh g^–1^, *n* = 300	5000 mA g^–1^, 500 mAh g^–1^		135
MnO_2_ nanosheets	Nickel foam	1000 mA g^–1^, 900 mAh g^–1^, *n* = 200			151
Graphene quantum dots–anchored VO_2_ nanobelts	Graphene foam	18000 mA g^–1^, ≈200 mAh g^–1^, *n* = 1500	36000 mA g^–1^, 151 mAh g^–1^	0.6	139
VO_2_(B)‐HMB nanoflakes	Graphene foam	1500 mA g^–1^, 305 mAh g^–1^, *n* = 500	9000 mA g^–1^, 219 mAh g^–1^	0.8	140
V_2_O_5_/PEDOT nanobelts	Graphene foam	1500 mA g^–1^, 265 mAh g^–1^, *n* = 500	18000 mA g^–1^, 163 mAh g^–1^	0.7	141
V_2_O_5_ nanoflake arrays	Carbon cloth	150 mA g^–1^, 275 mAh g^–1^, *n* = 100	3000 mA g^–1^, 181 mAh g^–1^	0.9	142
Co_3_O_4_ nanoblades	Nickel foam	445 mA g^–1^, 981 mAh g^–1^, *n* = 100	8900 mA g^–1^, 381 mAh g^–1^		143
CoO nanosheets	Nickel foam	1000 mA g^–1^, 1000 mAh g^–1^, *n* = 100	10000 mA g^–1^, 560 mAh g^–1^		150
CoO@C nanowires	Nickel foam	100 mA g^–1^, 1120 mAh g^–1^, *n* = 600	4000 mA g^–1^, 536 mAh g^–1^	1.7	144
CuO nanowires	Copper foam	0.15 mA cm^–2^, 687.4 mAh g^–1^, *n* = 120			145
CuO/C nanowires	Nickel foam	337 mA g^–1^, 610 mAh g^–1^, *n* = 290	2022 mA g^–1^, 360 mAh g^–1^	2	146
NiO nanorods	Nickel foam	1000 mA g^–1^, 705.5 mAh g^–1^, *n* = 70	5000 mA g^–1^, 575 mAh g^–1^	1.24–1.86	147
NiO nanosheets	Nickel foam	144 mA g^–1^, 1043 mAh g^–1^, *n* = 80	7180 mA g^–1^, 305 mAh g^–1^	1.32	149
NiO nanosheet arrays	Carbon cloth	700 mA g^–1^, 758.1 mAh g^–1^, *n* = 100	5000 mA g^–1^, 298.4 mAh g^–1^	1.77	153
ZnCo_2_O_4_ nanoarrays	Carbon cloth	180 mA g^–1^, 1180 mAh g^–1^, *n* = 100	1800 mA g^–1^, 750 mAh g^–1^	2.2–3.6	158
ZnCo_2_O_4_ nanowire arrays	Ni foam	100 mA g^–1^, 1050 mAh g^–1^, *n* = 60	2778 mA g^–1^, 240 mAh g^–1^	1.06–1.92	159
NiCo_2_O_4_ nanowrie arrays	Carbon cloth	500 mA g^–1^, 854 mAh g^–1^, *n* = 100	3000 mA g^–1^, ≈600 mAh g^–1^	1.2	160
NiCo_2_O_4_ nanowire arrays	Carbon cloth	500 mA g^–1^, 1085.5 mAh^–1^, *n* = 100	4000 mA g^–1^, 507 mAh g^–1^	1.3–1.5	161
MnCo_2_O_4_ nanosheet arrays	Carbon cloth	800 μA cm^–2^, 3 mAh cm^–2^, *n* = 60	1600 μA cm^–2^, 2 mAh cm^–2^	3	162
FeCo_2_O_4_ nanoneedle arrays	Nickel foam	100 mA g^–1^, 1335 mAh g^–1^, *n* = 200	2000 mA g^–1^, 875 mAh g^–1^	1.68	163
TiO_2_@SnO_2_ nanotubes	Nickel foam	1600 mA g^–1^, 580 mAh g^–1^, *n* = 50	3200 mA g^–1^, 498 mAh g^–1^		167
SnO_2_‐in‐TiO_2_ nanoarrays	Carbon cloth	400 mA g^–1^, 393.3 mAh g^–1^, *n* = 1000	3200 mA g^–1^, 241.2 mAh g^–1^	4.15	168
CuO/Co_3_O_4_ nanowires	Nickel foam	200 mA g^–1^, 1191 mAh g^–1^, *n* = 200	2500 mA g^–1^, 580 mAh g^–1^	2.5	172
CuO/CoO nanotubes	Copper foam	1000 mA g^–1^, 1078 mAh g^–1^, *n* = 1000	4000 mA g^–1^, 342 mAh g^–1^	2.6	173

Based on the current status of the developments in self‐supported metal oxide nanoarray electrodes for LIBs, we propose several important trends for the future research in this field. The first research direction lies in the electrode architecture design. The architectural design of electrodes is an effective method to achieve high energy density, power density, cycling stability, and safety for practical LIBs. An ideal electrode material should own the characteristics of high porosity, good electronic conductivity, light weight, and high stability.^188^, ^189^ These key criterions are more important in lithium‐ion micro‐batteries, which require the integration of high energy density and power density in small areas.^127^, ^190^ Light‐weight 3D graphene foams hold many virtues as advanced 3D substrates for loading nanoarrays, but their large porosity and poor mechanical strength are unfavorable. In this regard, 3D carbon cloth‐based electrodes with less open‐pore space, higher tap density and good mechanical stability are attractive.^49^, ^191^ For self‐supported metal oxide nanoarrays, the rate performance is greatly enhanced but the cycling performance is still unsatisfactory, especially for metal oxides with large volume expansion. Coating protective layers (such as carbon or conductive polymer) on the surface of nanoarrays with tight adhesion represents a useful strategy to improve the cycling stability. Alternatively, the introduction of a protective layer of another mtetal oxide into nanoarrays to form “yolk–shell” nanoarrays is an effective way to achieve better cycling stability.^168^, ^192^ Furthermore, there is an underlying safety problem associated with the high surface area of metal oxide nanoarray electrodes because the side reaction of the electrodes could be accelerated, resulting in the formation of an unstable SEI layer for metal oxides with large volume changes. The introduction of a suitable coating layer on the nanoarrays may be able to protect the SEI layer from cracking, thus improving the safety of the nanoarray electrodes.

The second direction is to develop cathodic nanoarray electrodes. The thin film electrodes of LiCoO_2_, LiMn_2_O_4_, and LiFePO_4_ as cathodes in LIBs have realized commercial applications for several decades. Although the appealing features in terms of long longevity, high output voltage, stable potential platform, and high safety associated with the anode graphite are found in these commercial cathode materials, the capacity and rate performance of the conventional film electrodes can meet the requirement for their long‐term development. The fabrication of nanoarray cathodes provides an appealing way to improve the high‐rate performance but only limited progress has been achieved so far because of the difficulties in the fabrication of cathode metal oxide nanoarrays. Therefore, it is demanding to develop effective techniques to achieve the fabrication of nanoarrays of various cathode materials including the commercially available materials and the newly developed materials like layered lithium‐rich materials,^193^, ^194^ on conductive substrates.

The third direction would be the electrode architecture design in full‐cell configurations. In principle, an anode material should be compatible with the available cathode materials and a cathode material should be capable of accommodating the commercial anode materials like graphite in full‐cell tests. This principle is particularly tough for the construction of nanoarray‐based full cells because the different morphologies between the nanoarray electrodes and counter electrodes usually cause the configuration mismatch between the two electrodes, leading to the capacity loss and the decay of cycling performance. Moreover, as the lithium source of the whole battery, the cathode materials in full‐cell configuration may not supply sustainable lithium ions and the inevitable lithium consumption due to the side reaction undoubtedly gives rise to the descending electrochemical performance, especially under high current density. In this respect, searching for nanoarray electrodes with ample lithium source and good high‐rate performance is a crucial concern for full‐cell development.
